# The diversity of recent trends for chondrichthyans in the Mediterranean reflects fishing exploitation and a potential evolutionary pressure towards early maturation

**DOI:** 10.1038/s41598-019-56818-9

**Published:** 2020-01-17

**Authors:** Sergio Ramírez-Amaro, Francesc Ordines, Antonio Esteban, Cristina García, Beatriz Guijarro, Francisca Salmerón, Bàrbara Terrasa, Enric Massutí

**Affiliations:** 1 Instituto Español de Oceanografía, Centre Oceanogràfic de les Balears, Moll de Ponent s/n, 07015 Palma, Spain; 2 Laboratori de Genètica, Universitat de les Illes Balears, 07122 Palma de Mallorca, Spain; 3 Instituto Español de Oceanografía, Centro Oceanográfico de Murcia, Varadero 1, 30740 San Pedro del Pinar, Murcia Spain; 4 Instituto Español de Oceanografía, Centro Oceanográfico de Málaga, Muelle Pesquero s/n, 29640 Fuengirola, Málaga Spain

**Keywords:** Ocean sciences, Marine biology

## Abstract

Chondrichthyans are a vulnerable group that has been overexploited for almost half a century in the Mediterranean. Since in this area most chondrichthyans are rarely incorporated into international statistics, the impact of fishing on their populations is difficult to assess. Here, we evaluate temporal trends in order to understand the recent history of chondrichthyans in the western Mediterranean. Fishery-independent data were obtained from scientific surveys carried out from 1994 to 2015 in three geographical sub-areas. Our results reflect fairly stable populations in terms of diversity, with some increase in density and standardized biomass of some species dwelling on the continental shelf, and even for some species dwelling on the slope. In contrast, decreasing trends were observed in some deep-water species. This can be explained by the reduction of the trawling effort on the continental shelf over the last few decades, and the shift of the fleet towards deep waters, along with the greater resilience displayed by some species. Furthermore, a decreasing trend in maturity of *Scyliorhinus canicula* was detected, suggesting an evolutionary response to overfishing. These results improve scientific knowledge for developing true adaptive management in the Mediterranean that will implement measures to strengthen or initiate the recovery of chondrichthyans.

## Introduction

**Figure 1:**
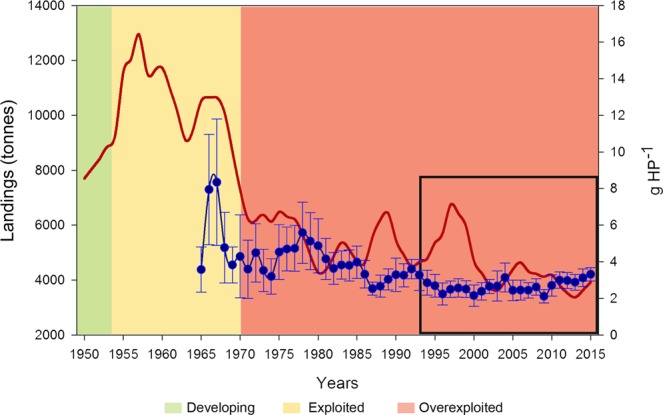
Historical series of landings of chondrichthyans (red line) in the Mediterranean and Black Sea according to FAO statistics^85^, and mean annual landings per unit effort (blue line; with standard error bars) of elasmobranchs in the Balearic Islands^24^. Colours correspond to different stages of the catches identified according to the algorithm for catch-based stock status^86^: green is “developing”, yellow is “exploited” and red is “overexploited”. The black square indicates our study period.

Marine ecosystems are under pressure from overfishing, habitat degradation and climate change which have altered the populations of most of their species^1,2^, especially chondrichthyans (sharks, rays and chimaeras), due to their low population resilience^3–5^. Global chondrichthyan landings, reported to the Food and Agriculture Organization of the United Nations (FAO), increased over 200% from 1950 to the peak year in 2003 and subsequently dropped almost 15% by 2011^6^. In the Mediterranean, the temporal evolution of chondrichthyan landings has gone through three stages: a development phase at the beginning of the 1950s; followed by intense fishing pressure from the mid 1950s to 1970, corresponding to the exploited phase; and a decreasing trend from 1970 to recent years, indicating an overexploited status (Fig. 1), such as has been reported for most demersal and pelagic bony fish and shellfish stocks a decade later during the eighties^7–10^. This earlier overexploitation in chondrichthyans is to be expected due to their low resilience. Studies of long time series of chondrichthyan catches conducted in several areas of the Mediterranean have also shown decreasing trends over the last few decades^11–14^. Furthermore, there is evidence that Mediterranean chondrichthyans have declined in diversity. At present, several species such as *Dipturus batis* (recently split into two cryptic species, *D*. cf. *flossada* and *D*. cf. *intermedia*^15^, *Pristis* spp. and *Squatina* spp.) are considered to be locally extinct^16–18^. In fact, the International Union for Conservation of Nature (IUCN) has recently revealed the Mediterranean as a key hotspot of extinction risk for chondrichthyans, where more than half of the species assessed (39 out of 73) are considered to be threatened: 20 critically endangered, 11 endangered and 8 vulnerable^19^.

In the western Mediterranean, most chondrichthyans are bottom dwelling species inhabiting demersal ecosystems on the continental shelf and slope, which makes them especially vulnerable to trawling^18^, the most important fishery in the western Mediterranean in terms of fishing capacity of both the fleet and catches^20^. This fishery operates over a wide bathymetric range (50–800 m), exploiting different communities^21^ and thus involving a large number of species in the catches, which include bony fishes, decapod crustaceans, cephalopods and other invertebrates, as well as chondrichthyans, which represent an important fraction of by-catch and discards^22–24^.

Ecosystem-based assessment and management of fisheries requires moving from the conventional single-species approach to entire exploited communities^25^. This is particularly important in the bottom trawling taking place along the western Mediterranean, with a marked multi-species character, and where by-catch species can show comparable or even higher levels of overexploitation than the target ones^26^, or are especially vulnerable to fishing exploitation, such as chondrichthyans^27^. However, a lack of accurate fishing landing data concerning chondrichthyans, and a paucity of biological information has been a drawback for the assessment of their populations^28^ and the Mediterranean is no exception. In this sense, fishery-independent data from scientific surveys can provide valuable information on by-catch species (e.g. abundance and biomass, sex and maturity, size composition) that is not commonly collected in fishery monitoring programs, which mainly focus on achieving data for assessing the most important target species.

Long-term trends of chondrichthyans have been studied in different areas of the Mediterranean, at either community^8,13,16,24,29^ or population level^30–33^. However, none of these studies have evaluated temporal changes over a broad area, combining community and species level. Our aim was to use fishery-indepent data from scientific surveys over the last few decades, to evaluate spatio-temporal patterns and trends in diversity, density standardized biomass, size and maturity of demersal chondricthyans in the western Mediterranean.

## Results

A total of 34 chondrichthyan species belonging to 13 families were caught throughout the western Mediterranean over the last two decades, 27, 26 and 19 species of which were caught in GSA01, GSA05 and GSA06, respectively (Table 1).

**Table 1:** Chondrichthyans captured in each geographical sub-area (GSA) considered by the General Fisheries Commission for the Mediterranean throughout the study area (1: GSA01 or Northern Alboran Sea; 5: GSA05 or Balearic Islands; 6: GSA06 or Northern Spain) during the MEDITS surveys conducted along the western Mediterranean during the periods 1994–2015 for GSA01 and GSA06 and 2001–2015 for GSA05.

Family	Species	D (m)			%Y			%D			N			B			IUCN	
		1	5	6	1	5	6	1	5	6	1	5	6	1	5	6	2007	2016
Chimaeridae	*Chimaera monstrosa*	325–831	440–654	440–856	95.4	53.3	50	55.3	5.6	4.2	1871	29	25	1407	2.25	1.95	NT	NT
Scyliorhinidae	*Galeus melastomus*	301–831	145–755	232–788	100	100	100	94.8	93.5	83.6	84127	45050	17340	17253	1539	1960	LC	LC
	*Galeus atlanticus*	324–821	—	—	93.7	—	—	41.7	—	—	3238	—	—	1007	—	—	NT	NT
	*Scyliorhinus canicula*	40–664	38–626	42–661	100	100	100	73.4	95.5	51.7	9634	82441	27945	1876	5701	3853	LC	LC
Triakidae	*Galeorhinus galeus*	238–593	—	—	59.1	—	—	8.4	—	—	38	—	—	528	—	—	VU	VU
	*Mustelus mustelus*	60	54–141	—	9.1	80	—	<0.1	11.2	—	4	207	—	4.4	80.4	—	EN	VU
	*Mustelus asterias*	—	107	—	—	13.3	—	—	—	—	—	2	—	—	1.2	—	EN	VU
Hexanchidae	*Heptranchias perlo*	428–556	—	—	9.1	—	—	<0.1	—	—	2	—	—	8.3	—	—	VU	DD
Centrophoridae	*Centrophorus granulosus*	377–796	594–753	—	81.8	33.3	—	10.7	3.2	—	52	9	—	184	36.35	—	VU	CR
Dalatiidae	*Dalatias licha*	325–831	590–743	411–748	100	86.6	63.6	15.8	4.4	5.2	124	8	23	500	19.24	8.7	DD	VU
Etmopteridae	*Etmopterus spinax*	314–831	362–755	236–856	100	100	100	88.7	58	41.2	8546	882	983	874	59.57	61.24	LC	LC
Oxynotidae	*Oxynotus centrina*	87–812	98	—	68.2	6.6	—	0.23	—	—	30	2	—	88.35	0.25	—	CR	CR
Squalidae	*Squalus acanthias*	77	104–654	124–582	4.5	46.6	9.1	—	4.6	2.1	1	53	56	1.5	65.6	13.46	EN	EN
	*Squalus blainvillei*	—	47–667	124–130	—	73.3	13.6	—	12.1	3.5	—	185	134	—	778	118.55	EN	DD
Dasyatidae	*Dasyatis centroura*	—	58	—	—	6.6	—	—	—	—	—	1	—	—	4.4	—	NT	VU
	*Dasyatis pastinaca*	—	39–98	54–79	—	100	13.6	—	47.3	<0.1	—	364	5	—	169.5	22.29	NT	VU
	*Pteroplatytrygon violacea*	43–317	—	—	18.2	—	—	<0.1	—	—	5	—	—	52.9	—	—	NT	LC
Myliobatidae	*Mylibatis aquila*	44	39–144	73–81	4.5	80	13.6	—	29.2	<0.1	2	200	8	0.61	322	24.32	NT	VU
	*Pteromylaeus bovinus*	—	44–60	—	—	6.6	—	—	<0.1	—	—	2	—	—	7.57	—	EN	CR
Rajidae	*Dipturus nidarosiensis*	765–813	—	—	9.1	—	—	<0.1	—	—	2	—	—	0.62	—	—	NE	NE
	*Dipturus oxyrinchus*	357–769	134–699	—	9.1	100	—	<0.1	32.3	—	2	509	—	9.82	315	—	NT	NT
	*Leucoraja circularis*	354–710	131–499	245	36.4	46.6	4.5	<0.1	4.7	—	13	139	1	269.5	29	0.043	EN	CR
	*Leucoraja naevus*	60–565	52–540	82–320	81.8	100	59	17.2	40.9	3.5	287	969	94	39.28	236	38.92	NT	NT
	*Raja asterias*	40–180	—	40–255	81.8	—	95.5	2.4	—	10.2	90	—	270	114.47	—	138	NT	NT
	*Raja brachyura*	—	48–162	—	—	33.3	—	—	23.2	—	—	417	—	—	95.87	—	DD	NT
	*Raja clavata*	47–420	57–590	52–582	18.2	100	84.4	0.16	64.7	4.3	16	4994	289	11.06	2369	20.9	NT	NT
	*Raja miraletus*	81–207	40–174	85–225	9.1	100	50	<0.1	38.9	1.2	3	2044	49	0.53	115.1	13.02	LC	LC
	*Raja montagui*	44–160	—	57–406	22.7		72.7	0.24		5.7	11		114	6.33		32.96	LC	LC
	*Raja polystigma*	70	48–424	85–316	4.5	94.7	31.8	—	33.1	<0.1	1	1826	39	0.79	266.7	12.53	NT	LC
	*Raja radula*	40–53	40–97	124–211	9.1	100	13.6	<0.1	35.2	<0.1	3	664	9	1.98	257	2.22	DD	EN
	*Rostroraja alba*	—	56–251	—	—	40	—	—	<0.1	—	—	13	—	—	71.2	—	CR	EN
Torpedinidae	*Torpedo marmorata*	40–290	51–258	45–297	100	86.6	100	26.4	5.2	21.2	187	71	253	116.8	9.5	71	LC	LC
	*Torpedo nobiliana*	170–777	368	144–431	81.8	6.6	22.7		—	<0.1	56	1	14	61.2	0.22	13.17	DD	LC
	*Torpedo torpedo*	40	—	—	13.6	—	—	—	—	—	11	—	—	6.92	—	—	LC	LC

D: depth range; %Y: average percentage of years in which the species were caught during the study period; %D: percentage of appearance within the depth range in which the species was caught for all years; N: total number of specimens caught; B: total weight of catches. IUCN indicates the regional status of species from the International Union for the Conservation of the Nature regional assessments performed in 2007 (Cavanagh and Gibson, 2017) and 2016 (Dulvy *et al*.^19^): DD (Data Deficient); LC (Least Concern); NT (Near Threatened); VU (Vulnerable); EN (Endangered); CR (Critically Endangered), not evaluated (NE).

### Diversity and species composition

**Figure 2:**
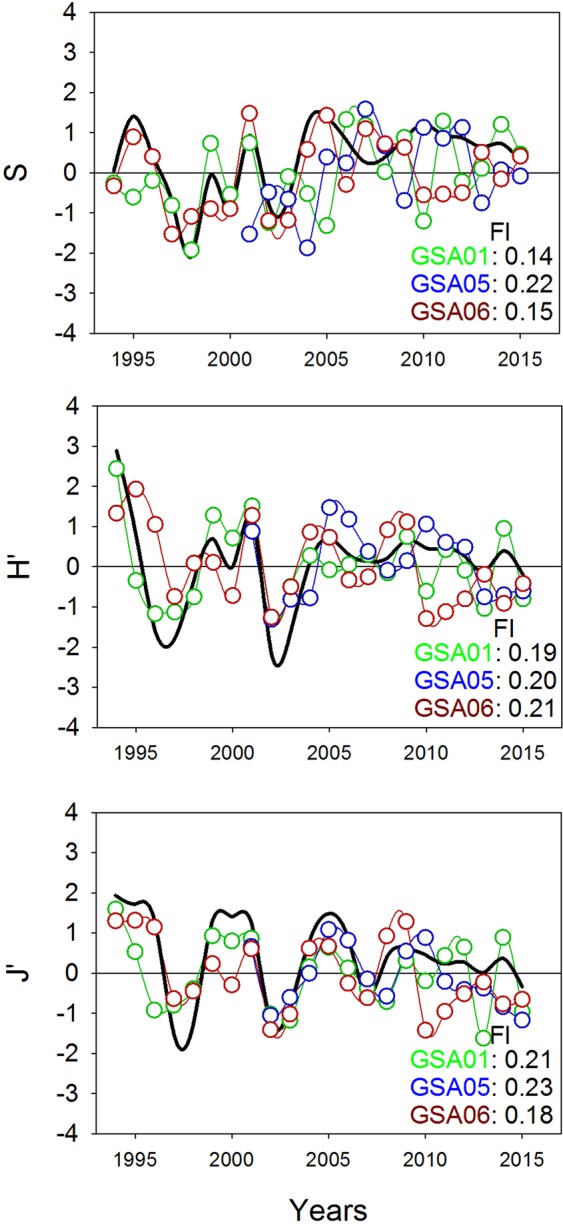
Common temporal trends (black lines) from dynamic factor analysis (DFA) applied to species richness (S), Shannon diversity (H’) and evenness (J’) per geographical sub-area (GSA) considered by the General Fisheries Commission for the Mediterranean in the study area (GSA01: Northern Alboran Sea, as green lines; GSA05: Balearic Islands, as blue lines; GSA06: Northern Spain, as red lines) during the periods 1994-2015 in GSA01 and GSA06, and 2001–2015 in GSA05. Factor loading (Fl) results for each GSA are indicated in the bottom right corner of each graph.

Time series exhibited similar trends for each diversity index (S, H’ and J’), with important fluctuations during the first 15 years, followed by stability over the last five years (Fig. 2, Supplementary Figure S1). DFA results of diversity indices are given in Supplementary Table S2.

*Scyliorhinus canicula* and *Galeus melastomus* were the most important sharks, and even chondrichthyans, in all GSAs. *Scyliorhinus canicula* represented >90% and >70% in terms of density and standardized biomass, respectively, in bathymetric strata B and C. In stratum D, *G*. *melastomus* corresponded to >50% in both density and standardized biomass in GSA01 and GSA05, while in GSA06, the most frequent was *S*. *canicula* (54% and 82% in density and standardized biomass, respectively). In stratum E, *G*. *melastomus* represented >85% in both density and standardized biomass (Supplementary Table S3).

The most important batoids in both density and standardized biomass showed differences not only by depth strata, but also by GSA (Supplementary Table S4). In GSA01, the most frequent species were *Torpedo marmorata* (>40% in both density and standardized biomass) and *Raja asterias* (14% and 55%, respectively) in stratum B, whilst *Leucoraja naevus* predominated in strata C and D (>70% in both density and standardized biomass). In GSA05, the most important species in stratum B were *Raja miraletus* (25% and 5% in density and standardized biomass, respectively), *Raja radula* (19% and 13%, respectively), and *Raja clavata* (19% and 34%, respectively); while *R. clavata* (56% and 78%, respectively), *Raja polystigma* (18% and 4%, respectively) and *L. naevus* (17% and 12%, respectively) predominated in stratum C; and *R. clavata* (59% and 62%, respectively) and *Dipturus oxyrinchus* (29% and 28%, respectively) predominated in stratum D. In GSA06, *R. clavata* predominated in all depth strata (≥28% and ≥40% in density and standardized biomass, respectively), while the second most important species was *R. asterias*, with ≥22% in both density and standardized biomass in stratum B, ≥9% in stratum C, and 33% and 15%, respectively, in stratum D.

### Density and standardized biomass

**Figure 3:**
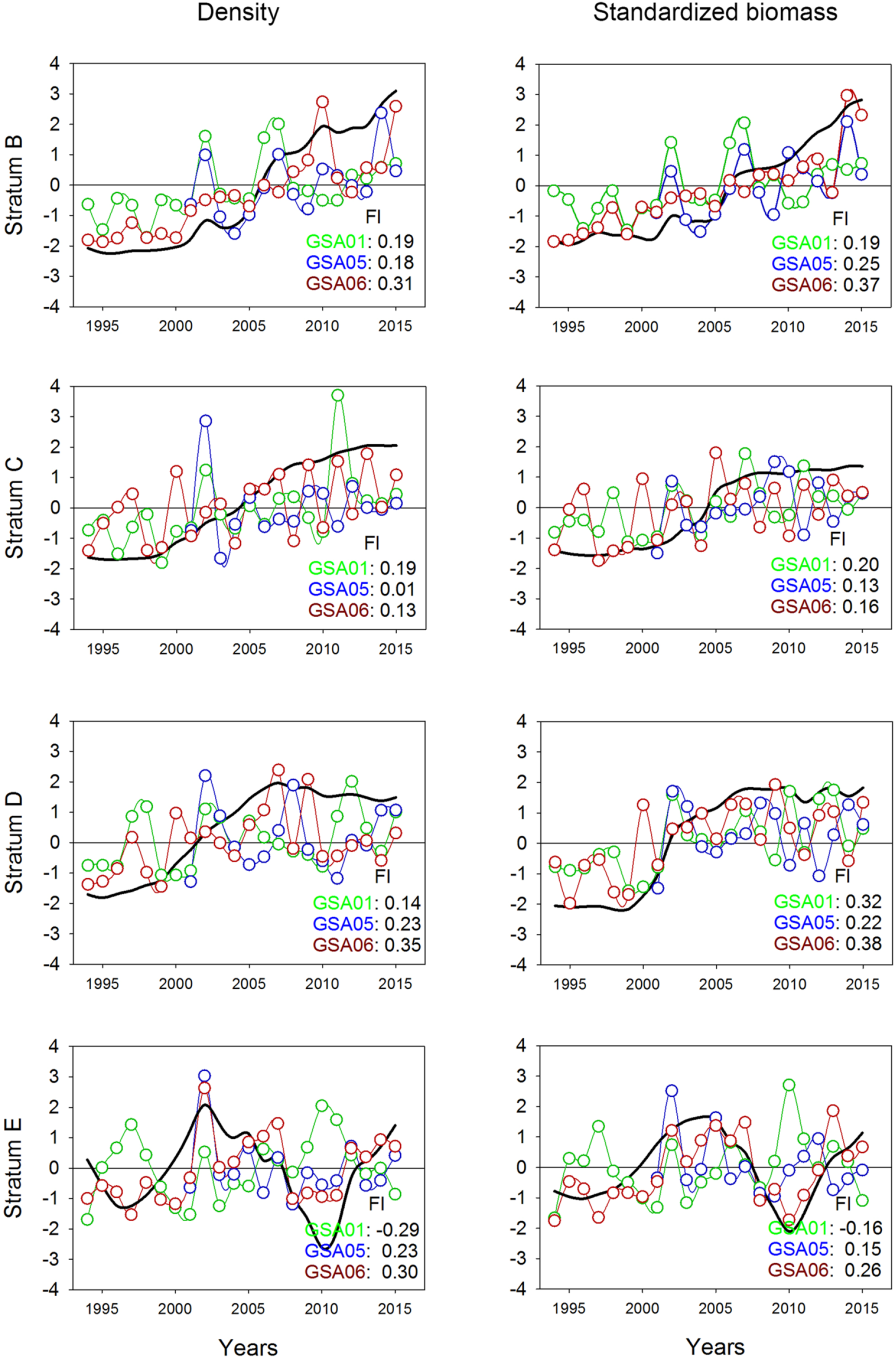
Common temporal trends (black lines) from dynamic factor analysis (DFA) applied to density and standardized biomass of sharks per geographical sub-area (GSA) considered by the General Fisheries Commission for the Mediterranean in the study area (GSA01: Northern Alboran Sea, as green lines; GSA05: Balearic Islands, as blue lines; GSA06: Northern Spain, as red lines) and depth stratum (B: 50–100 m; C: 101–200 m; D: 201–500 m; E: 501–800 m) during the periods 1994–2015 in GSA01 and GSA06, and 2001–2015 in GSA05. Factor loading (Fl) results for each GSA are indicated in the bottom right corner of each graph.

Time series of density and standardized biomass of sharks showed an increase over the study period in depth strata B, C, and D for the three GSAs. In stratum C, low factor loading values were observed in GSA05 and GSA06, suggesting stability over the study period. Stratum E exhibited cyclic changes throughout the study period. In this stratum, negative factor loadings were observed in the case of GSA01, indicating an inverse trend compared to the common trend (Fig. 3, Supplementary Figure S2).

**Figure 4:**
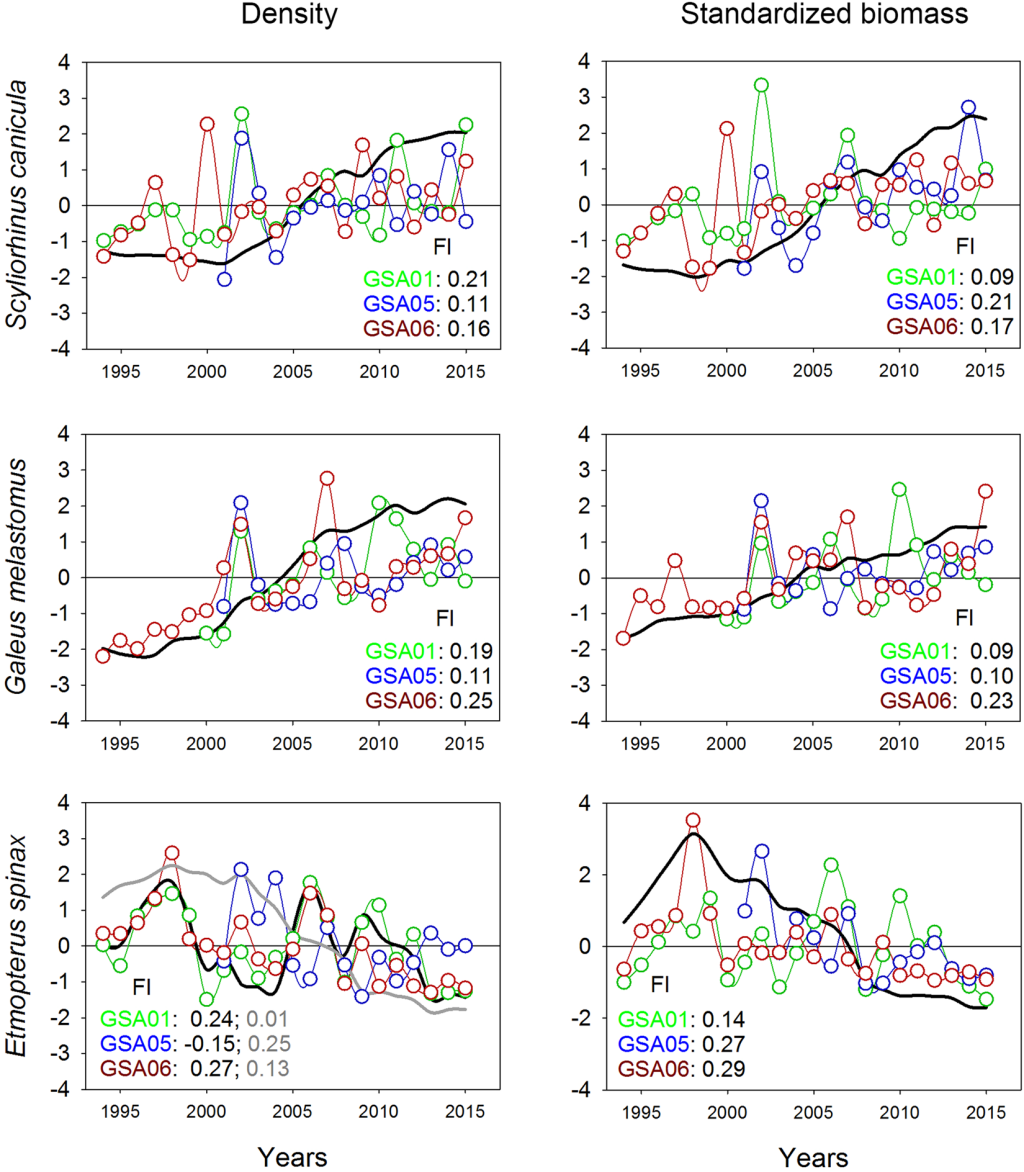
Common temporal trends (black lines) from dynamic factor analysis (DFA; grey lines correspond to the second common trend identified) applied to density and standardized biomass for the most abundant sharks, *Scyliorhinus canicula*, *Galeus melastomus* and *Etmopterus spinax*, per geographical sub-area (GSA) considered by the General Fisheries Commission for the Mediterranean in the study area (GSA01: Northern Alboran Sea, as green lines; GSA05: Balearic Islands, as blue lines; GSA06: Northern Spain, as red lines) during the periods 1994–2015 in GSA01 and GSA06, and 2001–2015 in GSA05. Factor loading (Fl) results for each GSA are indicated in the bottom corner of each graph.

The two most frequent species of sharks, *S*. *canicula* and *G*. *melastomus*, also showed increasing common trends in both density and standardized biomass over the last 10 years for all GSAs. Nonetheless, low factor loadings observed in density and standardized biomass for GSA05 and GSA02 respectively, suggesting stability throughout the whole period analysed (Fig. 4, Supplementary Figure S3). In the case of *Etmopterus spinax*, DFAs revealed two possible common trends in density, and one in standardized biomass. The trend in standardized biomass and second trend in density (Fig. 4) showed a decrease over the last 10 years for GSA05. In GSA01 and GSA06, the first trend in density exhibited stability, with fluctuations throughout the study period. DFA results for sharks are given in Supplementary Tables S5–S6.

**Figure 5:**
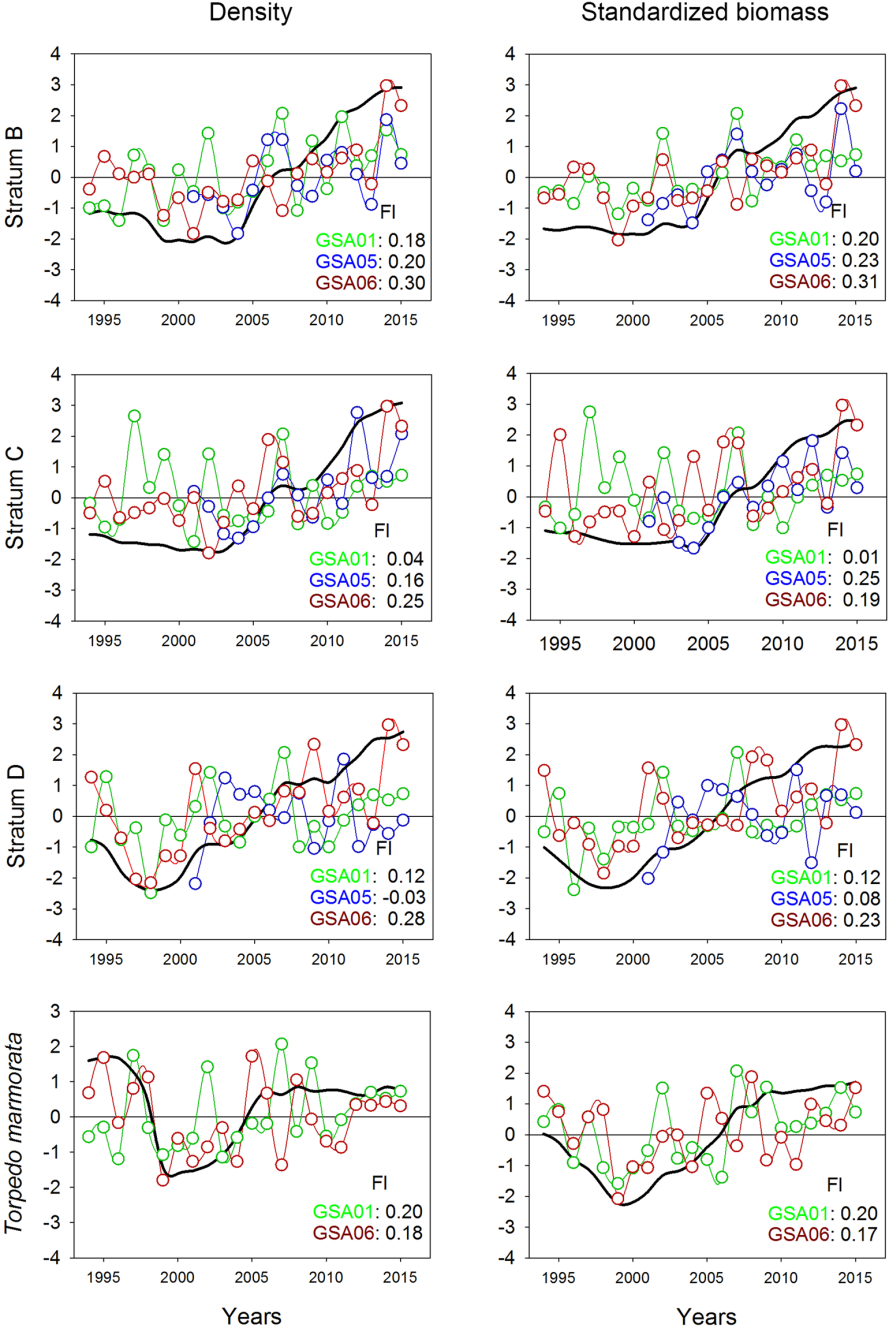
Common temporal trends (black lines) from dynamic factor analysis (DFA) applied to density and standardized biomass for batoids and *Torpedo marmorata*. For batoids, the three geographical sub-areas (GSAs) considered by the General Fisheries Commission for the Mediterranean in the study area (GSA01: Northern Alboran Sea, as green lines; GSA05: Balearic Islands, as blue lines; GSA06: Northern Spain, as red lines) and depth stratum (B: 50–100 m; C: 101–200 m; D: 201–500 m; E: 501–800 m) were utilized during the periods 1994–2015 in GSA01 and GSA06 and 2001–2015 in GSA05. For *Torpedo marmorata*, the GSA01 and GSA02 were considered. Factor loading (Fl) results for each GSA are indicated in the bottom right corner of each graph.

**Figure 6:**
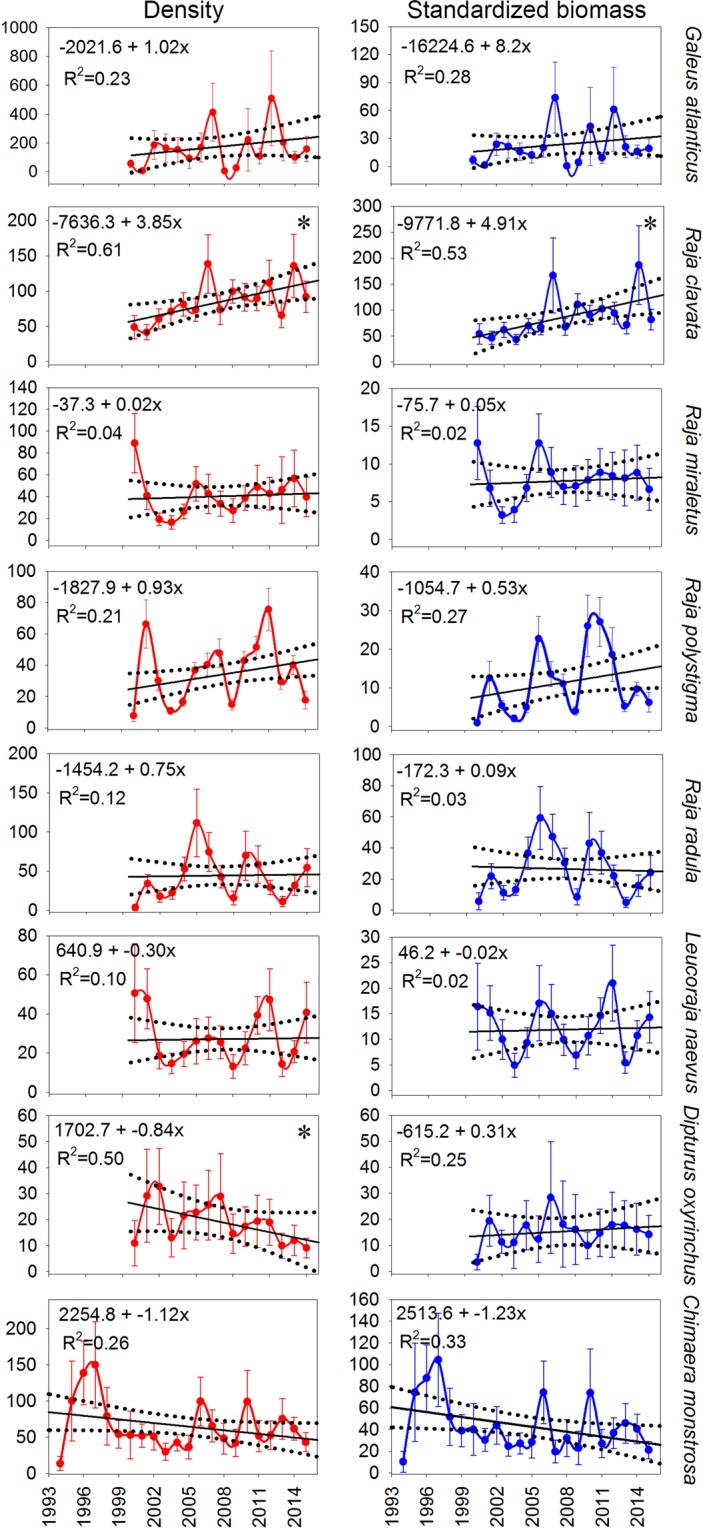
Linear regression models of density (in red) and standardized biomass (in blue) indices for the most important species within a particular GSA. *Galeus atlanticus* and *Chimaera monstrosa* in GSA01 (Northern Alboran Sea) during the periods 2000–2015 and 1994–2015, respectively. *Raja clavata*, *Raja miraletus*, *Raja polystigma*, *Raja radula*, *Leucoraja naevus* and *Dipturus oxyrinchus* in GSA05 (Balearic Islands) during the period 2001–2015. Dashed lines show 95% confidence intervals for regression lines, while asterisks indicate statistical significance: *p < 0.05; **p < 0.01; ***p < 0.001.

In general, batoids also showed a fairly common increasing temporal trend for the three GSAs, with a rise in density and standardized biomass over the last 10 years (Fig. 5, Supplementary Figure S4). GSA01 and GSA05 showed low factor loadings in strata C and D, respectively, exhibiting stability with fluctuations throughout the study period. *Torpedo marmorata* showed a decrease in both density and standardized biomass over the first five years, followed by an increase for the next eight years, and finally stability over the last five years (Fig. 5). DFA results for batoids are given in Supplementary Tables S7–S8. In GSA05, the most frequent batoid species, *R*. *clavata*, *R*. *miraletus*, *R*. *polystigma*, *R*. *radula*, *L*. *naevus* and *D*. *oxyrinchus*, exhibited different patterns (Fig. 6). *Raja clavata* showed an increase over the study period in both density and standardized biomass, while *R*. *miraletus*, *R*. *polystigma*, *R*. *radula* and *L*. *naevus* revealed stability and *D*. *oxyrinchus* showed a decrease in density, but stability for standardized biomass.

*Galeus atlanticus* and *Chimaera monstrosa* showed stability in both density and standardized biomass (Fig. 6). The first species was only present in GSA01, while the second one was also caught regularly at this GSA.

### Size

DFA models of size structure could be analysed for three species of sharks, *S*. *canicula*, *G*. *melastomus* and *E*. *spinax*. The results of DFA models of these species are given in Supplementary Tables S9–S11. In the case of batoids, the only species whose size structure could be analysed was *R. clavata* in GSA05.

**Figure 7:**
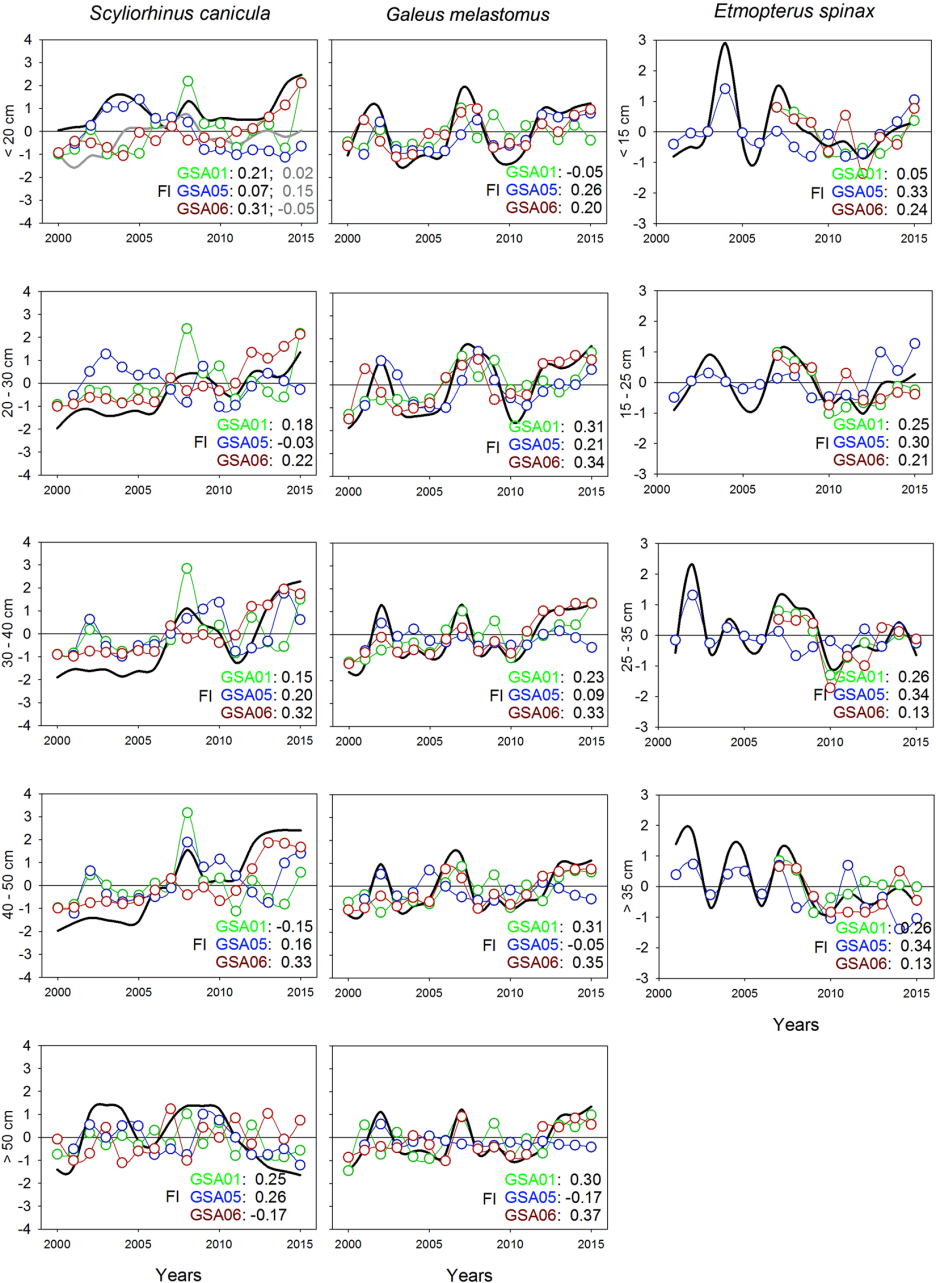
Common temporal trends (black lines) from dynamic factor analysis (DFA; grey lines correspond to the second common trend identified) applied to density by length category of *Scyliorhinus canicula*, *Galeus melastomus* and *Etmopterus spinax* per geographical sub-area (GSA) considered by the General Fisheries Commission for the Mediterranean in the study area (GSA01: Northern Alboran Sea, as green lines; GSA05: Balearic Islands, as blue lines; GSA06: Northern Spain, as red lines). The length categories of *S. canicula* and *G. melastomus* were the same. Factor loading (Fl) results for each GSA are indicated at the bottom right corner of each graphic.

For *S. canicula*, five length categories were modelled. In the category <20 cm TL, two common trends were observed. The first one was observed in GSA01 and GSA06, showing fluctuations over the early years, followed by stability in the last five years. The second one was observed in GSA05, with stability throughout the study period. The other categories (20–30, 30–40, 40–50 and >50 cm TL) showed a common trend, which suggests stability, with a slight increase in the last four years in the categories 30–40 and 40–50 (Fig. 7, Supplementary Figure S5).

The same length categories were analysed for *G*. *melastomus*. The categories <20 and 20–30 cm TL showed similar trends, with fluctuations in density across the study period, and a slight increase over the last five years. The remaining categories (30–40, 40–50 and >50 cm TL), in general showed stability with slight fluctuations over the study period. In the last two categories, GSA05 had a low negative factor loading, indicating a stability over the study period (Fig. 7, Supplementary Figure S5).

In *E*. *spinax*, four length categories were analysed. The category <15 cm TL showed a common trend with a sharp drop followed by a slight increase until 2007 and then stability until the end of the series. The category 15–25 cm TL showed slight increases and decreases. The category 25–35 cm TL exhibited a sharp drop until 2003 followed by apparent stability, with slight fluctuations after 2004. The category >35 cm TL showed cyclic changes, apparently every two years until 2009, followed by stability over the last five years (Fig. 7, Supplementary Figure S5).

**Figure 8:**
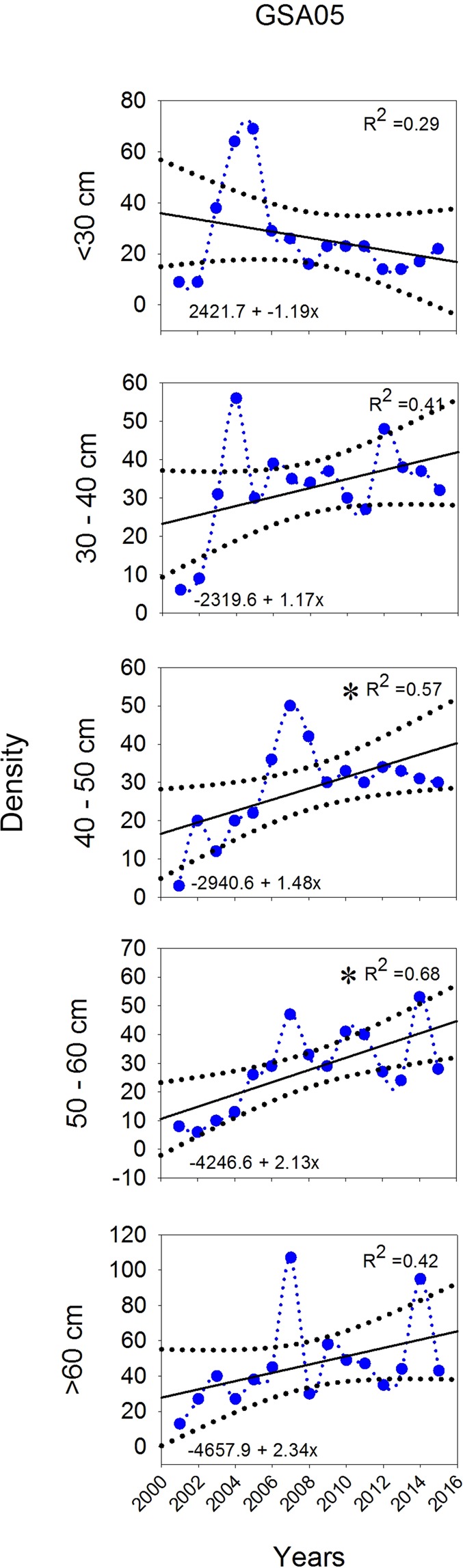
Linear regression models applied to density by length category of *Raja clavata* in GSA05 (Balearic Islands) during the period 2001–2015. Dashed lines show 95% confidence intervals for regression lines, while asterisks indicate statistical significance: *p < 0.05; **p < 0.01; ***p < 0.001.

In the case of *R*. *clavata*, five length categories were analysed. The categories 40–50 and 50–60 cm TL showed increasing trends, while the remaining categories (<30, 30–40 and >60 cm TL) displayed stability over the study period (Fig. 8).

### Maturity

Length at first maturity (L_50_) could be estimated for two sharks *S. canicula* and *G*. *melastomus*, in all GSAs, and one batoid, *R*. *clavata*, in GSA05. Parameters of maturity ogives by year, species, and sex are given in Supplementary Tables S12–S14.

**Figure 9:**
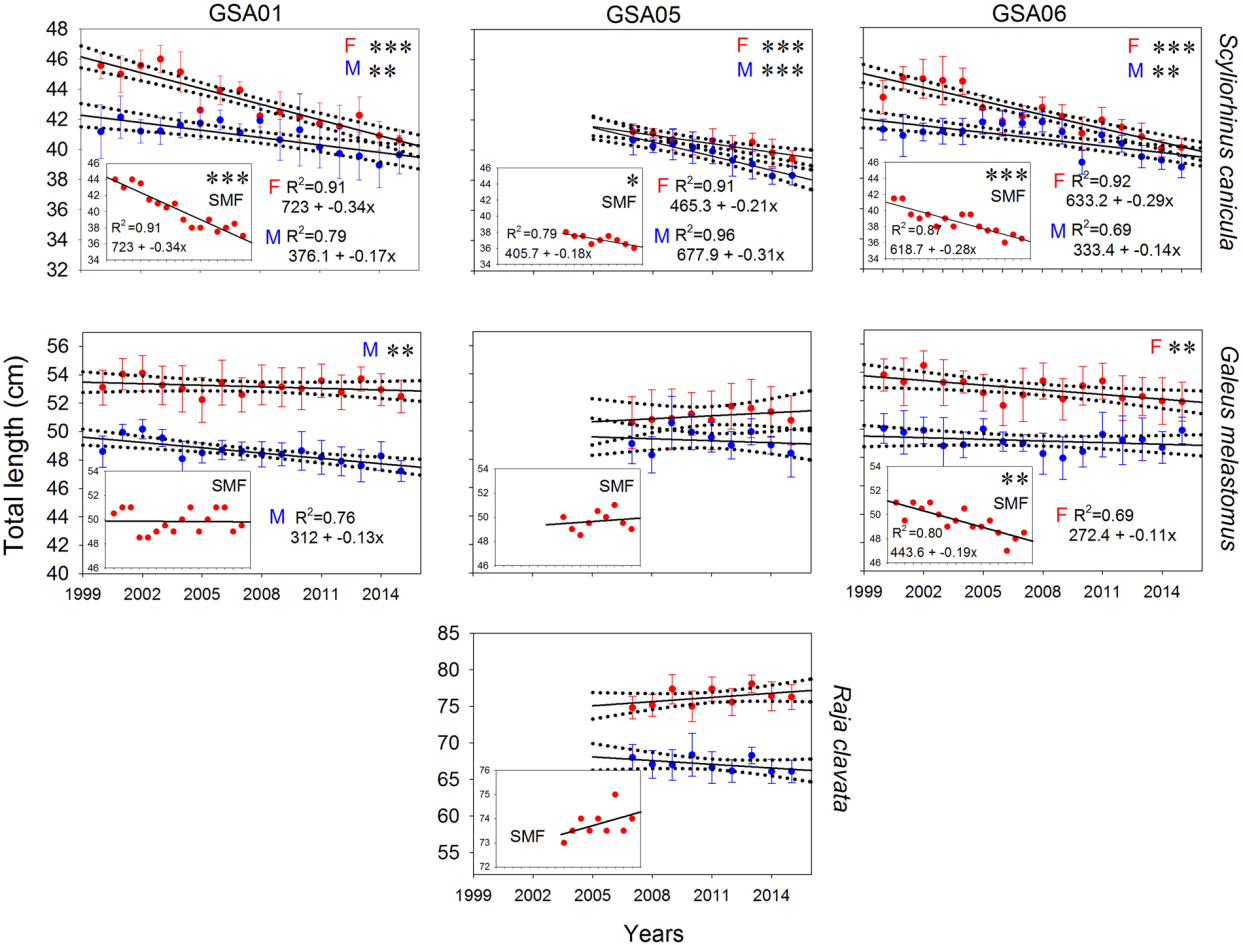
Linear regression models of mean annual length at first maturity (L_50_; standard deviation values are shown) for females (in red) and males (in blue) of *Scyliorhinus canicula* and *Galeus melastomus* per geographical sub-area (GSA) considered by the General Fisheries Commission for the Mediterranean in the study area (GSA01: Northern Alboran Sea; GSA05: Balearic Islands; GSA06: Northern Spain), and for *Raja clavata* in GSA05, during the periods 2000–2015 in GSA01 and GSA06 and 2007–2015 in GSA05. The SMF plots show the linear regression models for the smallest mature female. Dashed lines show 95% confidence intervals for regression lines, while asterisks indicate statistical significance: *p < 0.05; **p < 0.01; ***p < 0.001.

Linear regression models showed a decrease in L_50_ of *S. canicula* for both sexes and in all GSAs (Fig. 9). In females, this parameter decreased around 4 cm TL over 16 years in GSA01 and GSA06, and around 1.5 cm TL over 9 years in GSA05. For males this reduction was around 2 cm TL across the same periods. For *G*. *melastomus*, a decrease of L_50_ was also detected for females in GSA06 and males in GSA01, with a reduction of around 2 cm TL over 16 years in both sexes. By contrast, *R*. *clavata* did not show any trend in L_50_ over the study period. The size of the smallest mature female showed similar trends for the three species analysed (Fig. 9).

**Figure 10:**
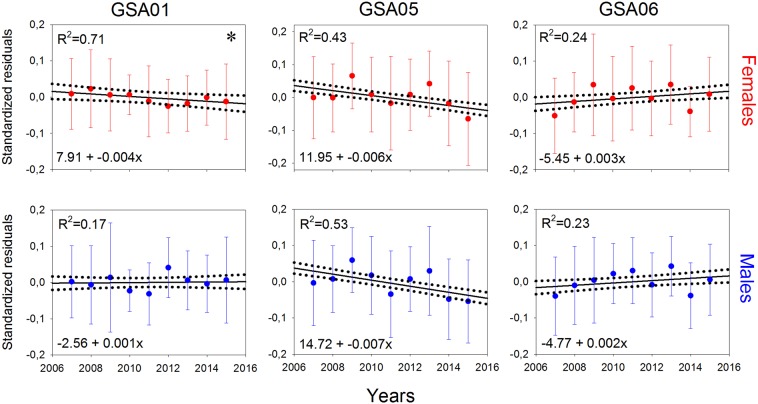
Linear regression models of somatic condition (mean and standard values) for females (in red) and males (in blue) of *Scyliorhinus canicula* by geographical sub-area (GSA) considered by the General Fisheries Commission for the Mediterranean in the study area (GSA01: Northern Alboran Sea; GSA05: Balearic Islands; GSA06: Northern Spain) during the period 2007–2015. Dashed lines show 95% confidence intervals for regression lines, while asterisks indicate statistical significance: *p < 0.05; **p < 0.01; ***p < 0.001.

Since *S. canicula* was the only species with a significant reduction in L_50_ in both sexes and in the three GSAs, its somatic factor was estimated for small-sized individuals (<35 cm TL). With the only exception of females in GSA01, no significant trends were found across the study period (Fig. 10).

## Discussion

In the Mediterranean, there has been increasing international concern for severe declines in the abundance and diversity of chondrichthyans, calling for urgent assessment of fishery exploitation. Here we applied a novel approach, at community and population level, to evaluate the temporal trends of demersal chondrichthyans in three geographical sub-areas along the western Mediterranean over the last two decades, using diversity, density, standardized biomass and biological parameters.

While the diversity indices used in this study measure different aspects of diversity, all of them exhibited similar trends, with relevant changes the firsts years (1994–2004) and stability the last 15 years for the three geographical sub-areas, such as has been observed in the diversity of demersal fishes^34^ and cephalopods^35^ throughout the whole Mediterranean. This diversity stability could be explained by the fact that relevant changes in diversity of demersal chondrichthyan communities may have occurred long before the onset of scientific monitoring of fisheries as has been reported in demersal fish communities^36^. Considering the available scientific literature in the Mediterranean^16,17,37–40^, there are differences in the demersal chondrichthyan community composition between early fishing explotation (1950’s) and the period studied here (1994–2015). In early fishing exploitation, some of the most threatened chondrichthyans currently such as *Galeorhinus galeus*, *Oxynotus centrina*, *Scyliorhinus stellaris*, *Squalus acanthias*, *Squatina* spp. and *Rhinobathos* spp.^18,19^ were common in descriptions of fish communities in the trawl fishing grounds off the Balearic Islands^37–40^ and the Adriatic Sea^17^. However, these species are no longer detected in scientific surveys for the assessment of demersal resources and have almost disappeared from commercial landings^16^, in some cases being considered locally extinct^41^. This change in chondrichthyan community can also be reflected in the percentage of species occurrence, wherein in early fishing exploitation period *Scyliorhinus canicula* and *Galeus melastomus* represented around 40% and 10%^16,17^ of species occurrence and its percentage of occurrence has recently increased to around 90 and 60%, respectively.

In contrast with the decreasing population trends described for chondrichthyan species in different areas of the Mediterranean, such as the Gulf of Lion^16^, the Ligurian Sea^13^, the Adriatic Sea^4,14^ and the Aegean Sea^12^, our results of density and standardized biomass suggested generally stable trends. Signs of recovery were even observed for the assemblages and the most abundant species inhabiting the continental shelf (*S*. *canicula*, *Torpedo mamorata* and *Raja clavata*) and for the most abundant species dwelling on the slope (*G*. *melastomus*). By contrast, decreasing trends were only detected for the deep water species, *Etmopterus spinax* and *Dipturus oxyrinchus*.

Fishing pressure could be one of the main factors contributing to the observed trends. The temporal evolution of the bottom trawl fleet in the western Mediterranean has gone through three phases^8,27^: (*i*) from the mid 1960s to mid 1970s, the number of vessels increased by a factor of 2.5; (*ii*) from the mid 1970s to 2000, this number decreased gradually, although the fishing capacity of this fleet remained constant or even increased, due to its continued growth in terms of engine power; and (*iii*) since 2000, the number of vessels has continued to decrease, probably along with the fishing capacity of the fleet, as mean engine power has remained fairly constant, especially during recent years. In this sense, our study covers a period of time when the chondrichthyan communities had already been altered in their diversity, and the effort of bottom trawl fishery was fairly stable or, at least, not increasing as previously. However, it is also necessary to consider the technological improvement of fishing gears and vessels during recent decades, which has enabled the worldwide expansion of fisheries to new areas, such as the open ocean and deep sea bottoms^42^. This has occurred in the western Mediterranean, where trawl fishery has moved from the continental shelf to the slope to capture the decapod crustaceans red shrimp (*Aristeus antennatus*) and Norway lobster (*Nephrops norvegicus*) with higher economic value^21,43^. The no increase in fishing effort of the bottom trawl, jointly with its displacement to deep waters, must be the basis for the current stability and recovery shown by sharks and batoids on the continental shelf. This shift in trawl activity has also been suggested to explain the recent stability observed in demersal chondrichthyans off Sardinia^44^, in the central western Mediterranean. Similarly, in the north-east Atlantic the progressive reduction of fishing pressure has resulted in the recovery of certain stocks, including *S*. *canicula*^45–47^.

The two most abundant demersal chondrichthyans in the western Mediterranean, *S*. *canicula* and *G*. *melastomus*, showed increasing trends despite their different bathymetric distribution (continental shelf and shelf break, and upper and middle slope, respectively^31,48^). Contrary to large chondrichthyans, these two small-medium sharks show life history traits that are less susceptible to fishing pressure (e.g. early maturity, shorter generation time, fast growth, continuous reproductive cycles^49–51^), giving them greater resilience and capacity of recovery^52^, as has been recently documented in small coastal sharks in the north-western Atlantic^53^. In addition, *S*. *canicula* and *G*. *melastomus* are considered to be opportunistic scavengers that may modify their natural diet to benefit from the disturbed sediments and discards generated by bottom trawling^54^; moreover, the former species has shown a high survival rate as a discarded catch of this fishery^55^. A similar situation has been observed for some batoids captured on the continental shelf (*R. brachyura*, *R*. *clavata*, *R. montagui* and *L. naevus*^56^), whose populations showed stability and even recovery in the present study.

The only decreasing trends were observed in the deep-water chondrichthyans, *D*. *oxyrinchus* and *E. spinax*. The decrease in the latter had already been reported in the north western Mediterranean^31,57^. Deep water chondrichthyans, with longer turnover times than the species dwelling on the continental shelf, are considered to be especially vulnerable to fishing exploitation^58,59^. This is the case of *E. spinax*, with late maturation, long reproductive cycle, low fecundity, and considerable longevity^60,61^. In this sense, *E. spinax* is quite different from *G. melastomus*, both species showing distinct life strategies and opposite trends. The deeper bathymetric distribution of *G. melastomus*, which in the western Mediterranean is relatively abundant down to 1400 m depth^62–64^, could also contribute to the stability of populations of this species. In this area, bottom trawl fishery does not reach more than 800–900 m depth^65^ and, moreover, the GFCM decided in 2006 to ban this fishery beyond 1000 m depth.

Regarding biological descriptors of main species, no clear trends were observed in size composition, although a decreasing trend in length at first maturity (L_50_) and the size of the smallest mature female was detected for *S*. *canicula* in all GSAs. It is known that fishing overexploitation could alter size structure and population parameters (e.g. fecundity, maturity, growth rate) on chondrichthyan populations in response to changes in species abundance (density-dependence change)^3,66–68^. Early maturity detected in *S*. *canicula* could be an indicator that the populations of this species have been under stress, due to the high level of trawl fishing exploitation in the study area. In fact, general overfishing of the demersal population around the Balearic Islands during the 1980s included elasmobranchs^8^. This early maturation, an adaptive response that increases the likelihood of offspring reaching maturity^69^, could have promoted the present recovery of *S*. *canicula*. Two possible mechanisms could explain this adaptation: an evolutionary response to selection for smaller size at maturity and/or by reducing intra-specific competition, due to reduction in species abundance, with the consequent increase in available food^66,70^. Environment is another potential mechanism that is deemed to influence life history changes in chondrichtyans^3,27^. Favourable conditions (e.g. intensive upwelling, high productivity) produce higher quality offspring that grow at faster rates, resulting in early maturation^67,68^. However, since no significant trends were found in somatic condition, this is not the case of *S*. *canicula* populations. Thus, the most plausible hypothesis to explain the early maturation of this species is its evolutionary response to overfishing. In fact, before changing growth rate, fishes use their somatic energy reserves to meet the running costs of their basal metabolism (e.g., maintenance, immune defence, and cognition), digestion, or routine activities such as migration to breeding grounds, courting, competition for mates and breeding sites, copulation, and parental care^71,72^.

Detecting changes in population parameters of chondrichthyans is difficult since they are typical k strategists in comparison to teleosts^3,73^. Given that small size chondrichthyans have a higher intrinsic population growth rate than larger species^52,73^, these changes have been detected mainly in few small-medium sharks such as *Rhizoprionodon terraenovae*^68^ and *Squalus acanthias*^74^. These change in population parameters could provide a compensatory mechanism in small-medium chondrichthyans decreasing the natural and fishing mortality, and likely promoting the recovery of those exploited populations^75^.

In summary, this study shows the feasibility of fishery-independent scientific surveys, which are currently being developed in the Mediterranean, to evaluate the populations trends of by-catch vulnerable species such as demersal chondrichthyans. Our trend models reveal that most of the sharks and batoids currently making up these communities have resisted the impact of fishing on the western Mediterranean over the last two decades. However, this is not the case for two deep water species, which are not resisting fishing exploitation. These results can be explained by the evolution of the trawl fishery (reduction of effort and displacement to deeper waters) over the last few decades, jointly with the great resilience displayed by some of these species, like *S*. *canicula*, a small-medium shark which also showed a reduction of its length at first maturity, probably as an evolutionary response to the general overfishing of the demersal populations exploited by bottom trawl fishery since the 1980s in the study area. Our findings, which come from a long period of over-exploitation of these vulnerable species - some of which appeared to be fairly common and have currently almost disappeared or are even considered to be locally extinct - provide a current baseline scenario in order to implement management measures that will strengthen or initiate the recovery of chondrichthyans in the Mediterranean, whose populations play an important role in marine ecosystems, and to develop true adaptive management in the area, making the sustainability of trawl fishery compatible with the conservation of marine ecosystems.

## Methods

### Ethic statement

Data were obtained in the framework of the MEDITS project and refer to the Mediterranean Iberian Peninsula and the Balearic Islands. The sampling scheme followed a standartized protocol^76^ approved by international authorities (EU/DG Mare, FAO/GFCM). If a live specimen of a rare species or a species subject to conservation measures was caught, it was quickly sampled (4–5 minutes) and returned back to the sea unharmed, giving it a chance for survival, following the recommendation GFCM/36/2012/3 (http://www.gfcmonline.org/decisions/) on fisheries management measures for conservation of sharks and rays in the GFCM area. In the cases the animal was alive when it arrived on the vessel during the scientific survey (MEDITS – DCF, EU Reg. 199/2008), it was suppressed by administering an overdose of anaesthetic in compliance with the recommendation of Decree Law n. 26 of 4 March 2014. All efforts were made to minimize suffering.

### Data source

**Figure 11:**
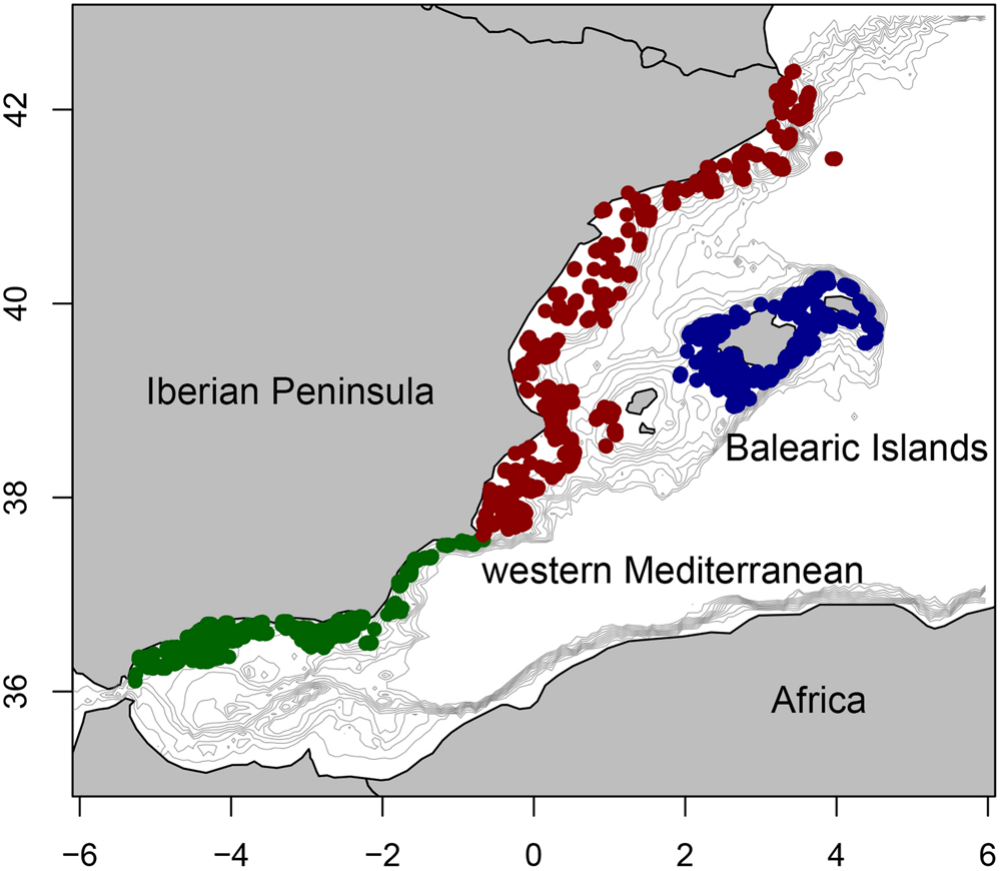
Map of the western Mediterranean showing the MEDITS stations developed between 1994 and 2015 used in the present study. Colours correspond to the three geographical sub-areas (GSAs) considered by the General Fisheries Commission for the Mediterranean in the study area: green is GSA01 (Northern Alboran Sea); blue is GSA05 (Balearic Islands) and red is GSA06 (Northern Spain). Isobaths between 50 and 2000 m (every 200 m) are also shown.

A total of 3158 stations were sampled during the MEDITS bottom trawl surveys, carried out annually during spring and early summer from 1994 along the Mediterranean Iberian Peninsula and the Balearic Islands (Fig. 11). The sampling method is described in MEDITS protocol^76^. A stratified random sampling scheme was applied, considering five bathymetric strata on the continental shelf and slope (A: 30–50 m; B: 51–100 m; C: 101–200 m; D: 201–500 m; E: 501–800 m) and the  three geographical sub-areas (GSAs) recognised by the General Fisheries Commission for the Mediterranean (GFCM) off the Spanish coast (GSA01: Northern Alboran Sea; GSA05: Balearic Islands; GSA06: Northern Spain; Fig. 2). The number of stations per year, GSA and bathymetric stratum are given in Supplementary Table S1. The sampling gear was the experimental bottom trawl GOC-73, equipped with a 20 mm mesh size in the cod-end. Hauls were conducted during daylight hours at a towing speed of around 3 knots, with an effective duration of 30’ at depths shallower than 200 m and 60’ below this depth. The arrival and departure of the net to the bottom, in addition to its horizontal and vertical openings (2.4–3 and 16–22 m, respectively) were measured using a SCANMAR system (http://www.scanmar.no/en/) consisting in a set of sensors attached to the gear, which allow its depth and geometry during the haul to be measured simultaneously.

The catch of each sample was sorted, identified to species level, counted and weighed, and length of individuals was measured. Data on abundance and biomass were standardized to number of individuals per km^2^ (referred to as “density”) and to kg per km^2^ (referred to as “standardized biomass”), respectively. To do so, the bottom surface sampled during the haul was considered, estimated from the horizontal opening of the net, and the distance covered, obtained from a GPS system. Sex and sexual maturity were determined from macroscopic examination of the gonads, following Holden and Raitt’s maturity scale^77^, which considers four stages for both females and males: immature, maturing, spawning and post-spawning.

The time series of data on density and standardized biomass spanned from 1994 to 2015 for GSA01 and GSA06, and from 2001 to 2015 for GSA05. Due to changes in the MEDITS sampling protocol over the years, data series on size and sexual maturity were shorter. In GSA01 and GSA06 it comprised the period 2000–2015, whereas available periods in GSA05 were 2001–2015 and 2007–2015 for size and sexual maturity, respectively.

### Data analysis

Dynamic factor analysis (DFA), a multivariable method applied to relatively short and non stationary time series data to detect common trends^77,78^, was applied to identify common trends between GSAs for diversity, density, standardized biomass and size structure parameters. In DFA, time series data are modelled as a linear combination of underlying common trends and factor loading indicates the strength of the influence of each GSA time series on the corresponding common trend. In order to achieve a proper interpretation of our results, the time series were standardized by subtracting the mean and dividing by the standard deviation^78^. Factor loadings with values above 0.20 (in absolute value) signifies that the corresponding GSA has a high contribution to the common trend detected^77^. Negative factor loading values indicate that the particular GSA has an inverse trend to the one detected as a common trend. Correlation of observation errors was modelled using different covariance matrices: (*i*) same variance and no covariance (diagonal-equal); (*ii*) different variances and no covariance (diagonal-unequal); (*iii*) same variance and covariance (equalvarcov); and (*iv*) different variances and covariances (unconstrained). The corrected Akaike information criterion (AICc) was used as a measure of goodness of fit, with the best model having the lowest AICc^78,79^. Model implementation was performed using the Multivariate Autoregressive State-Space “MARSS” package^80^ in R 3.3.2.

DFAs were applied to diversity, density and standardized biomass time series in order to search for common trends between GSAs along the study period. In this study, we evaluated the following diversity indices to identify a set of indicators that could provide a good representation of changes in demersal chondrichthyan communities, taking account the different aspects of diversity: mean species richness index (S), the Shannon-Wiener diversity index (H’) and evenness (J’) that were estimated by year and GSA.

The mean density and standardized biomass of the chondrichthyan groups (sharks, batoids and chimaeras) were calculated by year, GSA and depth stratum, except in stratum A, which was excluded from the analysis due to the low number of sampling stations. These parameters were also estimated by GSA for the most abundant species within their bathymetric distribution range. These species were those representing ≥85% density or standardized biomass in at least two GSAs, or with a percentage occurrence greater than 20% within their depth range of distribution for all GSAs. Since the presence of batoids was practically zero below 500 m depth, stratum E was not considered for this group.

Temporal trends in size structure were also evaluated for the most important species in the three GSAs. To do this, sexes were pooled into different categories according to the total length (TL) of the species, and the mean density by year was calculated. DFAs were applied to identify common trends between GSAs in density values for each length category from 2000 to 2015.

Meanwhile, linear regression analyses were applied to detect temporal trends for the most important species within a particular GSA (i.e. those representing ≥85% density and standardized biomass and with a percentage occurrence within the depth range of distribution ≥20%), in both density and size structure. *Dasyatis pastinaca* was not included in this analysis because of the possible misidentification with *Dasyatis* cf. *tortonesei* in the Balearic Islands^81^.

Linear regression analysis was also used to detect temporal trends of size at first maturity of the most important species. Maturity was assessed for species with at least 50 individuals sampled per year and GSA. To do this, maturation stages were converted into binary data (stages *i* and *ii* as immature and stages *iii* and *iv* as mature), while length at first maturity (L_50_; length at which 50% of the individuals are mature) was obtained by fitting a logistic regression to the proportion of mature individuals by length class. This was done for females and males and by year and GSA. The smallest length of mature females per year for each GSA was also estimated. In addition, considering the fact that the condition of fishes has been reported as an important factor affecting growth rate^82^ and that an increase in growth rate before the onset of maturation can lead to a decrease age and therefore size and maturity^83^, we also analysed the temporal trend in somatic condition for species with a significant decreasing trend in L_50_. For these species, we calculated the somatic condition for immature individuals (below the length at first maturity reported for these species in the study area^48^) of both sexes. Individual total weight (TW) and total length (TL) were log-transformed and the linear relationship between logTW and logTL was established by year. Residuals of the differences between the observed and predicted logTW were calculated and standardized, dividing each one by the standard deviation of their predicted values^84^. Lastly, linear regression analysis was applied to detect temporal trends from 2007 to 2015 in each GSA.

## Supplementary information

Supplementary information.
