# Supplementary material for: The diversity of recent trends for chondrichthyans in the Mediterranean reflects fishing exploitation and a potential evolutionary pressure towards early maturation
Source: Sci Rep. 2020 Jan 17;10:547. doi: 10.1038/s41598-019-56818-9 (PMC6969072; doi:10.1038/s41598-019-56818-9)
Supplement: Supplementary file 1 — Supplementary information. [file 41598_2019_56818_MOESM1_ESM.pdf]

Supporting information

The diversity of recent trends for chondrichthyans in the Mediterranean reflects fishing exploitation and a potential evolutionary pressure towards early maturation

Running title: trends in Mediterranean chondrichthyans

Sergio Ramírez-Amaro[1,2*], Francesc Ordines[1], Antonio Esteban[3], Cristina García[4], Beatriz Guijarro[1], Francisca Salmerón[4], Bàrbara Terrasa[2], Enric Massutí[1].

[1]*Instituto Español de Oceanografía, Centre Oceanogràfic de les Balears, Moll de Ponent s/n, 07015 Palma, Spain.*

[2]*Laboratori de Genètica, Universitat de les Illes Balears, 07122 Palma de Mallorca, Spain.*

[3]*Instituto Español de Oceanografía, Centro Oceanográfico de Murcia, Varadero 1, 30740 San Pedro del Pinar, Murcia, Spain.*

[4]*Instituto Español de Oceanografía, Centro Oceanográfico de Málaga, Muelle Pesquero s/n, 29640 Fuengirola, Málaga, Spain.*

Supplementary Figures

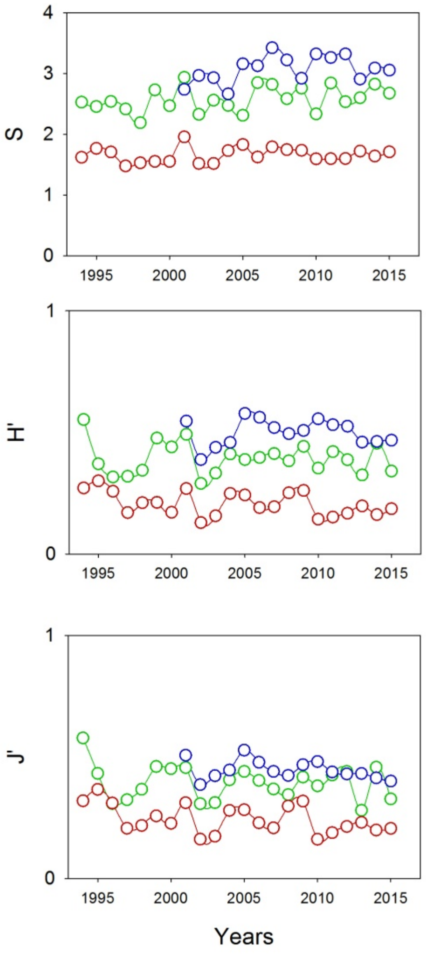

Figure S1. Time series of mean species richness (S), Shannon diversity (H') and evenness (J') per year and by geographical sub-area (GSA) considered by the General Fisheries Commission for the Mediterranean in the study area (GSA01: Northern Alboran Sea, as green lines; GSA05: Balearic Islands, as blue lines; GSA06: Northern Spain, as red lines) during the periods 1994-2015 in GSA01 and GSA06, and 2001-2015 in GSA05.

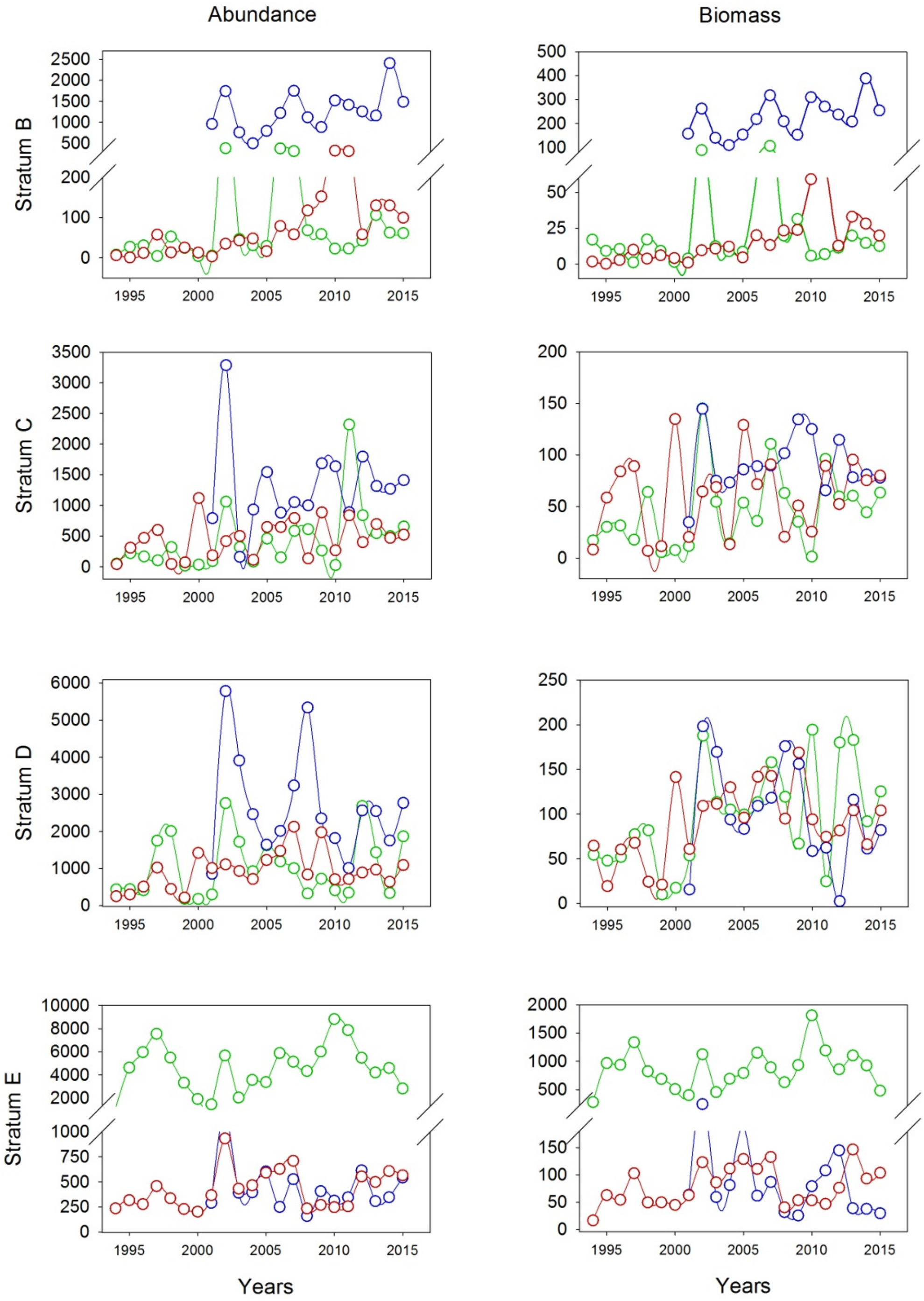

Figure S2. Time series of mean density and standardized biomass of sharks per geographical sub-area (GSA) considered by the General Fisheries Commission for the Mediterranean in the study area (GSA01: Northern Alboran Sea, as green lines; GSA05: Balearic Islands, as blue lines; GSA06: Northern Spain, as red lines) and depth stratum (B: 50-100 m; C: 101-200 m; D: 201-500 m; E: 501-800 m) during the periods 1994-2015 in GSA01 and GSA06, and 2001-2015 in GSA05.

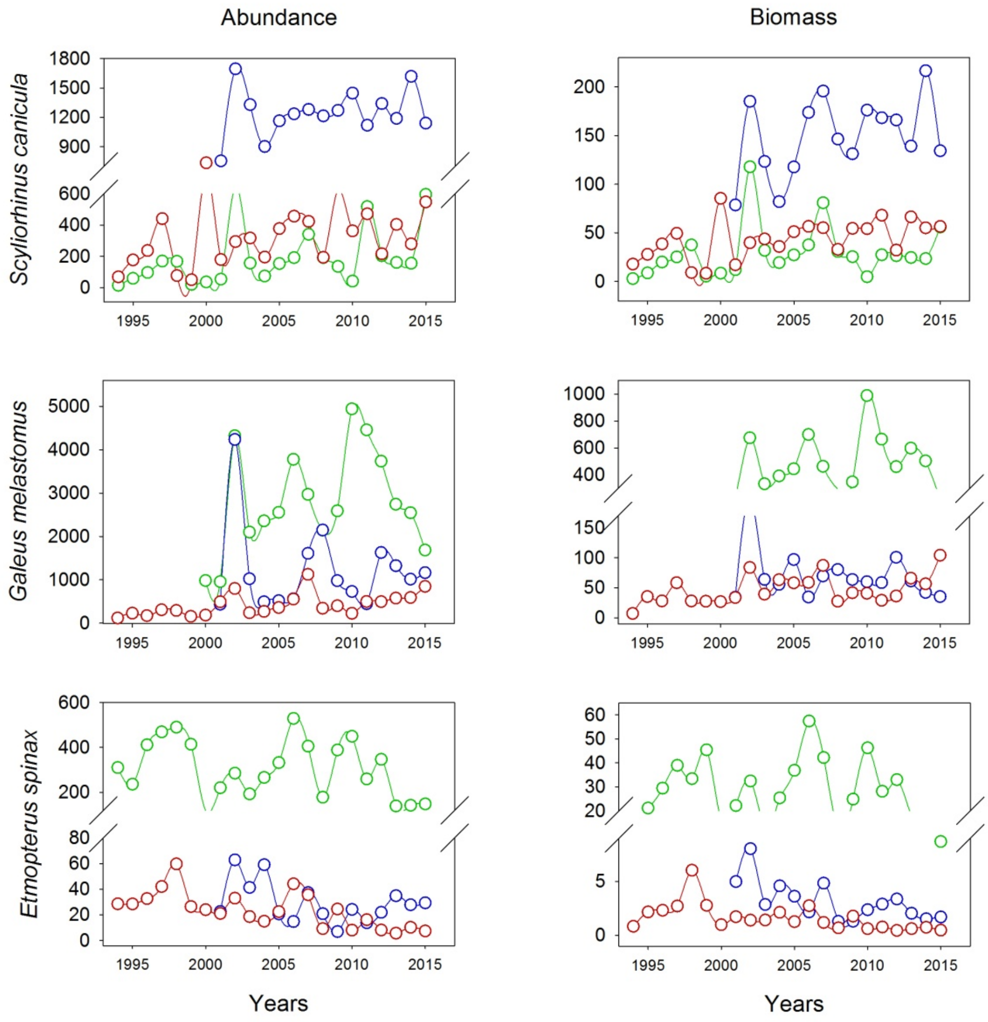

Figure S3. Time series of mean density and standardized biomass for the most abundant sharks, *Scyliorhinus canicula*, *Galeus melastomus* and *Etmopterus spinax*, per geographical sub-area (GSA) considered by the General Fisheries Commission for the Mediterranean in the study area (GSA01: Northern Alboran Sea, as green lines; GSA05: Balearic Islands, as blue lines; GSA06: Northern Spain, as red lines) during the periods 1994-2015 in GSA01 and GSA06, and 2001-2015 in GSA05.

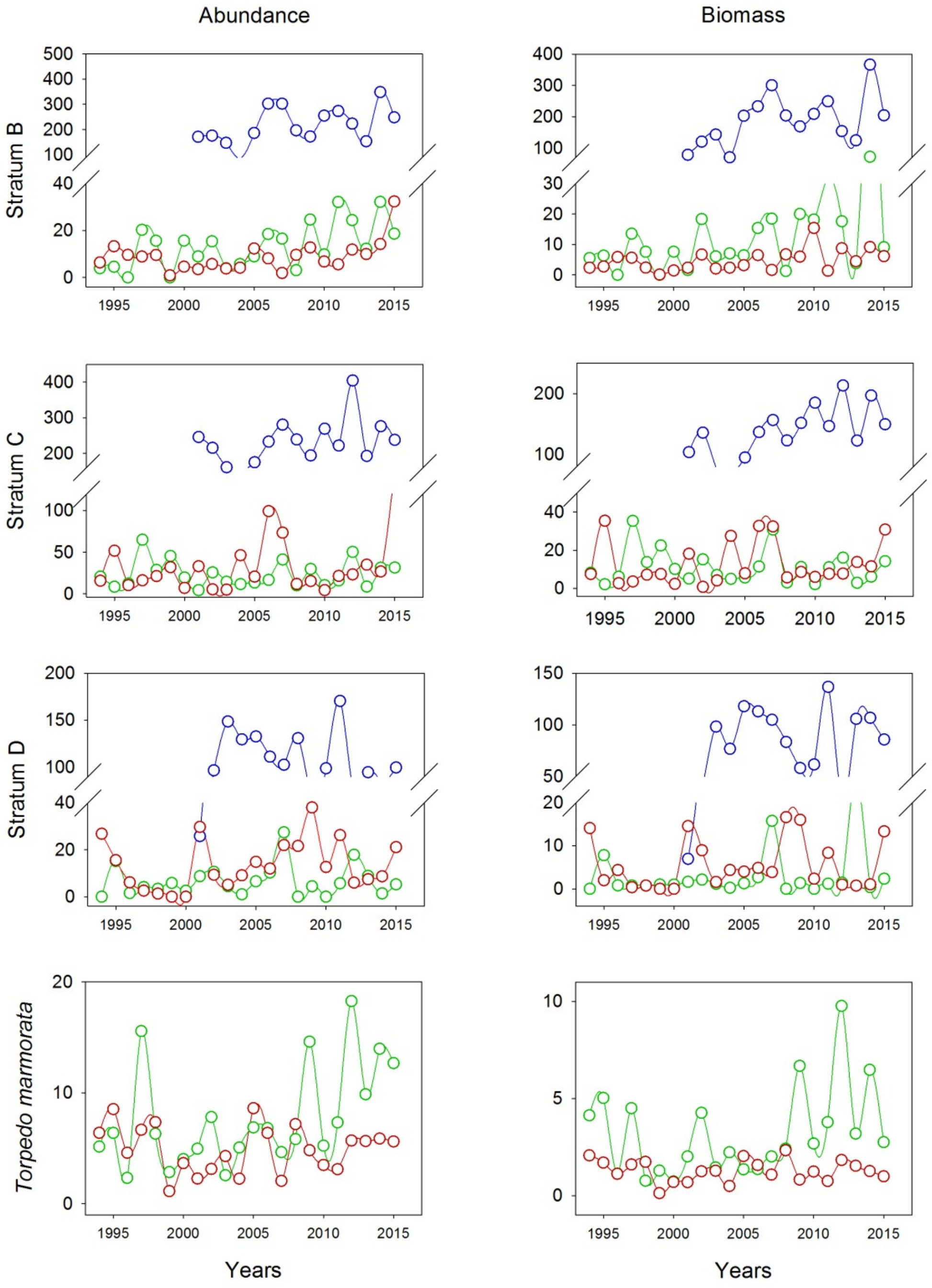

Figure S4. Time series of mean density and standardized biomass for batoids and *Torpedo marmorata*. For batoids, the three geographical sub-areas (GSAs) considered by the General Fisheries Commission for the Mediterranean in the study area (GSA01: Northern Alboran Sea, as green lines; GSA05: Balearic Islands, as blue lines; GSA06: Northern Spain, as red lines) and depth stratum (B: 50-100 m; C: 101-200 m; D: 201-500 m; E: 501-800 m) were utilized during the periods 1994-2015 in GSA01 and GSA06 and 2001-2015 in GSA05. For *Torpedo marmorata*, the GSA01 and GSA02 were considered.

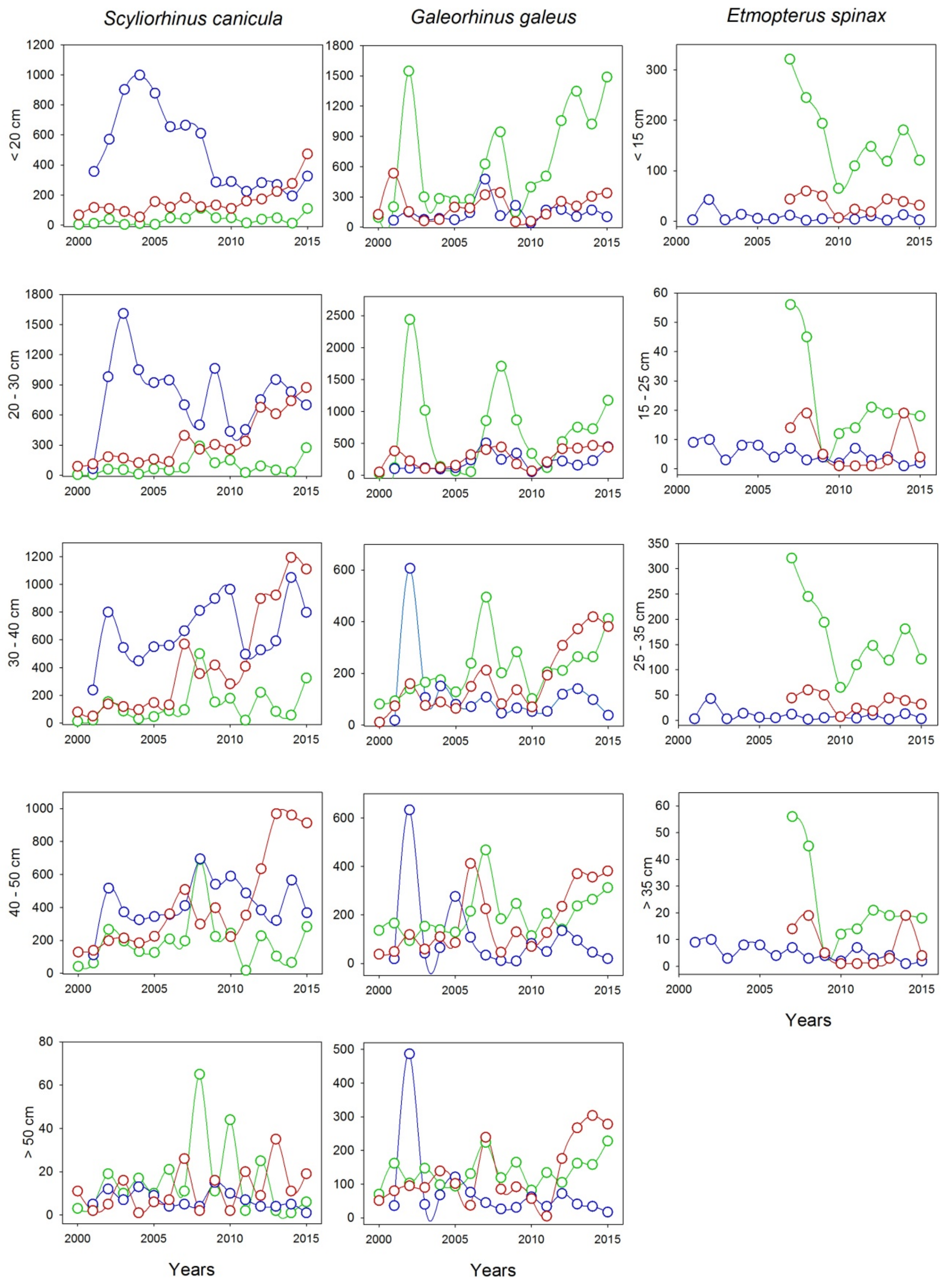

Figure S5. Time series of mean density by length category of *Scyliorhinus canicula*, *Galeus melastomus* and *Etmopterus spinax* per geographical sub-area (GSA) considered by the General Fisheries Commission for the Mediterranean in the study area (GSA01: Northern Alboran Sea, as green lines; GSA05: Balearic Islands, as blue lines; GSA06: Northern Spain, as red lines). The length categories of *S. canicula* and *G. melastomus* were the same.

Supplementary Tables

Table S1. Number of bottom trawl samples analysed by year, depth stratum (D) established in the MEDITS surveys (B: 50-100 m; C: 101-200 m; D: 201-500 m; E: 501-800 m) and geographical sub-area (GSA) considered by the General Fisheries Commission for the Mediterranean throughout the study area (GSA01: Northern Alboran Sea; GSA05: Balearic Islands; GSA06: Northern Spain) during the periods 1994-2015 for GSA01 and GSA06 and 2001-2015 for GSA05.

**GSA**	D	1994	1995	1996	1997	1998	1999	2000	2001	2002	2003	2004	2005	2006	2007	2008	2009	2010	2011	2012	2013	2014	2015
**GSA01**	B	5	5	5	6	6	9	6	6	8	12	8	8	7	8	7	7	6	6	8	11	13	14
	C	3	3	3	5	5	5	5	5	8	6	5	6	5	7	6	6	4	4	4	5	7	9
	D	6	9	11	10	7	11	12	10	11	11	13	11	11	13	11	11	6	8	9	10	14	19
	E	6	9	12	10	12	12	9	13	13	14	13	11	15	13	7	5	6	7	10	10	14	15
**GSA05**	B								12	23	20	27	23	29	20	20	20	20	21	20	22	22	22
	C								12	15	15	18	17	16	14	14	14	14	14	14	14	14	14
	D								8	10	11	12	9	10	8	8	8	8	9	8	9	11	8
	E								9	11	10	12	10	9	8	8	8	8	9	8	8	12	12
**GSA06**	B	19	25	26	25	27	26	29	29	34	36	30	31	33	26	28	28	19	28	34	38	37	30
	C	10	17	15	15	12	17	17	19	19	20	17	17	18	15	21	20	12	21	22	24	26	28
	D	9	15	13	11	8	13	11	20	18	18	17	16	22	13	14	14	10	18	16	19	16	20
	E	8	13	11	8	6	12	7	10	9	10	13	10	11	10	11	12	7	8	8	6	9	12

Table S2. Results from dynamic factor analysis (DFA) model fitting applied to species richness (S), Shannon diversity (H') and evenness (J') per haul of chondrichthyans per year during the periods 1994-2015 in GSA01 (Northern Alboran Sea) and GSA06 (Northern Spain) and 2001-2015 in GSA05 (Balearic Islands). For each model, the covariance matrix structure, the number of common trends (m) and the corrected Akaike Information Criterion (AICc) are given. Models in italics represent those selected as the models that produced the most favorable covariance matrix structure (with the lowest AICc values).

Length category	Covariance matrix structure	m	Log-likelihood	AICc
Richness (S)	*Diagonal and equal*	*1*	*-82.20*	*173.14*
	Equalvarcov	1	-82.16	175.44
	Diagonal and unequal	1	-82.08	177.79
	Diagonal and equal	2	-82.19	178.01
	Equalvarcov	2	-82.15	180.50
	Diagonal and equal	3	-82.20	180.59
	Unconstrained	1	-80.00	181.67
	Diagonal and unequal	2	-82.08	183.05
	Equalvarcov	3	-82.16	183.19
	Diagonal and unequal	3	-82.08	185.84
	Unconstrained	2	-80.00	187.62
	Unconstrained	3	-80.01	190.78
Diversity (H')	*Equalvarcov*	*1*	*-78.74*	*168.62*
	Equalvarcov	2	-78.72	173.69
	Diagonal and equal	1	-82.66	174.07
	Equalvarcov	3	-78.74	176.37
	Unconstrained	1	-78.24	178.16
	Diagonal and unequal	1	-82.29	178.21
	Diagonal and equal	2	-82.66	178.94
	Diagonal and equal	3	-82.67	181.52
	Diagonal and unequal	2	-82.29	183.47
	Unconstrained	2	-78.24	184.11
	Diagonal and unequal	3	-82.29	186.25
	Unconstrained	3	-78.25	187.27
Evenness (J')	*Diagonal and equal*	*1*	*-80.05*	*168.84*
	Equalvarcov	2	-76.92	170.03
	Equalvarcov	1	-80.50	172.13
	Diagonal and unequal	1	-79.49	172.60
	Equalvarcov	3	-76.92	172.72
	Diagonal and equal	2	-80.04	173.71
	Unconstrained	1	-76.41	174.49
	Diagonal and equal	3	-80.04	176.29
	Diagonal and unequal	2	-79.45	177.78
	Unconstrained	2	-76.40	180.44
	Diagonal and unequal	3	-79.45	180.57
	Unconstrained	3	-76.41	183.60

Table S3. Species composition for the most frequent species of sharks in terms of density (DE) and standardized biomass (BIO) captured in the MEDITS surveys throughout the western Mediterranean, by geographical sub-area (GSA) considered by the General Fisheries Commission for the Mediterranean in the study area (GSA01: Northern Alboran Sea; GSA05: Balearic Islands; GSA06: Northern Spain) and depth stratum (B: 50-100 m; C: 101-200 m; D: 201-500 m; E: 501-800 m) during the periods 1994-2015 for GSA01 and GSA06, and 2001-2015 for GSA05.

GSA	Species	Stratum B		Species	Stratum C		Species	Stratum D		Species	Stratum E	
		DE	BIO		DE	BIO		DE	BIO		ABU	BIO
**GSA01**	*Scyliorhinus canicula*	99.5	92.7	*Scyliorhinus canicula*	99.8	99.6	*Galeus melastomus*	77.1	76.1	*Galeus melastomus*	87.3	87.6
	Other	<0.5	<7.3	Other	<0.2	<0.4	*Scyliorhinus canicula*	10.4	9.9	*Etmopterus spinax*	8.1	4.5
							Etmopterus spinax	7.2	3.8	*Galeus atlanticus*	4.1	3.5
							Galeus atlanticus	4.8	3.9	Other	<0.5	<0.4
							Other	<0.5	<6.3			
**GSA05**	*Scyliorhinus canicula*	99.5	96.1	*Scyliorhinus canicula*	94.2	74.9	*Galeus melastomus*	63.2	51.6	*Galeus melastomus*	93.3	95.2
	Mustelus mustelus	0.3	3.8	*Squalus blainville*	5.72	24.9	*Scyliorhinus canicula*	36.3	45.1	*Etmopterus spinax*	6.3	4.12
	Other	<0.1	<0.1	Other	<0.1	<0.2	Other	<0.5	<3.1	Other	<0.4	<0.7
**GSA06**	*Scyliorhinus canicula*	100	100	*Scyliorhinus canicula*	97.9	90.3	*Scyliorhinus canicula*	54.6	81.8	*Galeus melastomus*	88	91.8
				Squalus acanthias	1.7	8.9	*Galeus melastomus*	44.7	17.8	*Etmopterus spinax*	7.7	3.5
				Other	<0.4	<0.8	Other	<0.7	<0.4	*Scyliorhinus canicula*	4	4.1
										Other	<0.3	<0.6

Table S4. Species composition for the most frequent species of batoids in terms of density (DE) and standardized biomass (BIO) captured in the MEDITS surveys throughout the western Mediterranean, by geographical sub-area (GSAs) considered by the General Fisheries Commission for the Mediterranean in the study area (GSA01: Northern Alboran Sea; GSA05: Balearic Islands; GSA06: Northern Spain) and depth stratum (B: 50-100 m; C: 101-200 m; D: 201-500 m) during the periods 1994-2015 for GSA01 and GSA06 and 2001-2015 for GSA05.

**GSA**	**Species**	**Stratum B**		**Species**	**Stratum C**		**Species**	**Stratum D**	
		DE	BIO		DE	BIO		DE	BIO
**GSA01**	*Torpedo marmorata*	45.1	14.3	*Leucoraja naevus*	71.7	82.1	*Leucoraja naevus*	86.1	72.9
	Raja asterias	43.3	54.9	*Torpedo marmorata*	23.9	11.6	*Torpedo nobiliana*	7.6	11.9
	Leucoraja naevus	3.8	2.9	*Raja asterias*	2	4.1	*Torpedo marmorata*	4.3	4.4
	Other	<7.8	<28	Other	<2.4	<2.2	Other	<2	<10.8
**GSA05**	*Raja miraletus*	24.6	5.4	*Raja clavata*	55.9	77.7	*Raja clavata*	58.7	61.8
	Raja radula	18.9	12.6	*Raja polystigma*	18.3	3.9	*Dipturus oxyrinchus*	28.7	27.7
	Raja clavata	18.9	34.5	*Leucoraja naevus*	16.7	12.1	*Leucoraja naevus*	7.2	5.1
	Raja polystigma	14.4	9.5	*Raja miraletus*	3.9	0.8	*Raja polystigma*	2.9	0.8
	Dasyatis pastinaca	4.9	9.5	Other	<0.6	<0.6	Other	<2.5	<4.5
	Raja brachyura	4.9	6.2						
	Other	<14	<23						
**GSA06**	*Raja clavata*	28.2	42.7	*Raja clavata*	30.2	39.6	*Raja clavata*	36.2	54.8
	Raja asterias	21.9	22.7	*Raja asterias*	11.6	9.2	*Raja asterias*	32.9	15.1
	Torpedo marmorata	20.2	9.8	*Torpedo marmorata*	9.8	4.3	*Leucoraja naevus*	12.5	11.6
	Leucoraja naevus	10	6.8	*Leucoraja naevus*	8.7	3.4	*Torpedo nobiliana*	3.8	2.5
	Raja montagui	5.3	3.9	*Raja miraletus*	7.7	3.2	Other	<14.6	<16
	Other	<14.4	<14.1	*Raja montagui*	5.2	3.5			
				Other	<26.8	<36.8			

Table S5. Results from dynamic factor analysis (DFA) model fitting applied to density (DE) and standardized biomass (BI) indices for sharks by depth stratum (B: 50-100 m; C: 101-200 m; D: 201-500 m; E: 501-800 m) during the periods 1994-2015 in GSA01 (Northern Alboran Sea) and GSA06 (Northern Spain) and 2001-2015 in GSA05 (Balearic Islands). For each model, the covariance matrix structure, the number of common trends (m) and the corrected Akaike Information Criterion (AICc) are given. Models in italics represent those selected as the models that produced the most favorable covariance matrix structure (with the lowest AICc values).

**Strata**	**Covariance matrix structure**		**m**		**Log-likelihood**		**AICc**	
	DE	BI	DE	BI	DE	BI	DE	BI
Stratum B	*Diagonal and equal*	*Diagonal and unequal*	*1*	*1*	*-77.49*	*-71.41*	*163.73*	*156.45*
	Equalvarcov	Diagonal and equal	1	1	-76.97	-74.22	165.07	157.18
	Diagonal and unequal	Equalvarcov	1	1	-76.45	-73.23	166.51	157.59
	Unconstrained	Unconstrained	1	1	-73.87	-69.75	169.43	161.18
	Equalvarcov	Diagonal and unequal	2	2	-76.97	-71.41	170.14	161.71
	Diagonal and equal	Diagonal and equal	2	2	-79.23	-74.21	172.09	162.05
	Diagonal and equal	Equalvarcov	3	2	-78.90	-73.22	174.01	162.65
	Unconstrained	Diagonal and unequal	2	3	-73.55	-71.41	174.73	164.50
	Equalvarcov	Diagonal and equal	3	3	-78.79	-74.21	176.46	164.63
	Diagonal and unequal	Equalvarcov	2	3	-79.11	-73.22	177.11	165.33
	Unconstrained	Unconstrained	3	2	-73.55	-69.75	177.89	167.12
	Diagonal and unequal	Unconstrained	3	3	-78.73	-69.64	179.14	170.29
Stratum C	*Diagonal and equal*	*Diagonal and equal*	*1*	*1*	*-82.02*	*-80.29*	*172.78*	*169.33*
	Equalvarcov	Equalvarcov	1	1	-81.42	-80.03	173.97	171.20
	Diagonal and unequal	Diagonal and unequal	1	1	-81.98	-80.23	177.57	174.07
	Diagonal and equal	Diagonal and equal	2	2	-82.02	-80.29	177.65	174.21
	Equalvarcov	Equalvarcov	3	2	-81.42	-80.03	179.03	176.27
	Diagonal and equal	Diagonal and equal	3	3	-82.02	-80.29	180.23	176.79
	Equalvarcov	Equalvarcov	3	3	-81.42	-80.04	181.72	178.95
	Diagonal and unequal	Diagonal and unequal	2	2	-81.98	-80.23	182.84	179.33
	Unconstrained	Unconstrained	1	1	-80.92	-78.94	183.52	179.55
	Diagonal and unequal	Diagonal and unequal	3	3	-81.98	-80.22	185.63	182.13
	Unconstrained	Unconstrained	2	2	-80.92	-78.94	189.47	185.50
	Unconstrained	Unconstrained	3	3	-80.91	-78.93	192.63	188.66
Stratum D	Diagonal and equal	*Diagonal and equal*	*1*	*1*	*-82.26*	*-77.41*	*173.28*	*163.56*
	Equalvarcov	Equalvarcov	1	1	-81.84	-77.16	174.82	165.46
	Diagonal and unequal	Diagonal and unequal	1	1	-82.19	-76.72	178.00	167.06
	Diagonal and equal	Diagonal and equal	2	2	-82.27	-77.41	178.15	168.44
	Equalvarcov	Equalvarcov	2	2	-81.84	-77.16	179.89	170.53
	Diagonal and equal	Diagonal and equal	3	3	-82.27	-77.41	180.73	171.02
	Equalvarcov	Diagonal and unequal	3	2	-81.85	-76.72	182.57	172.33
	Unconstrained	Equalvarcov	1	3	-82.65	-77.16	186.99	173.21
	Diagonal and unequal	Unconstrained	2	1	-86.68	-77.19	192.25	174.06
	Unconstrained	Diagonal and unequal	2	3	-82.57	-76.72	192.77	175.12
	Diagonal and unequal	Unconstrained	3	2	-86.68	-76.19	195.04	180.01
	Unconstrained	Unconstrained	3	3	-82.55	-76.20	195.89	183.17
Stratum E	*Equalvarcov*	*Equalvarcov*	*1*	*1*	*-74.80*	*-80.82*	*160.74*	*172.77*
	Unconstrained	Diagonal and equal	1	1	-70.39	-82.17	162.45	173.09
	Equalvarcov	Equalvarcov	2	2	-74.36	-80.56	164.92	177.32
	Unconstrained	Diagonal and equal	2	2	-70.76	-82.18	169.14	177.97
	Equalvarcov	Unconstrained	3	1	-76.11	-78.59	171.11	178.85
	Unconstrained	Diagonal and unequal	3	1	-70.65	-82.88	172.08	179.37
	Diagonal and equal	Equalvarcov	1	3	-82.30	-80.66	173.34	180.20
	Diagonal and unequal	Diagonal and equal	1	3	-80.88	-82.17	175.38	180.55
	Diagonal and unequal	Unconstrained	2	2	-78.41	-79.58	175.70	186.78
	Diagonal and equal	Diagonal and unequal	2	2	-81.79	-85.11	177.19	189.11
	Diagonal and equal	Unconstrained	3	3	-81.37	-79.73	178.94	190.24
	Diagonal and unequal	Diagonal and unequal	3	3	-78.92	-85.64	179.51	192.97

Table S6. Results from dynamic factor analysis (DFA) model fitting applied to density (DE) and standardized biomass (BI) indices for most abundant sharks, *Scyliorhinus canicula* (Sc), *Galeus melastomus* (Gm) and *Etmopterus spinax* (Es) during the periods 1994-2015 in GSA01 (Northern Alboran Sea) and GSA06 (Northern Spain) and 2001-2015 in GSA05 (Balearic Islands). For each model, the covariance matrix structure, the number of common trends (m) and the corrected Akaike Information Criterion (AICc) are given. Models in italic represent those selected as the models that produced the most favorable covariance matrix structure (with the lowest AICc values).

**Species**	**Covariance matrix structure**		**m**		**Log-likelihood**		**AICc**	
	DE	BI	DE	BI	DE	BI	DE	BI
Sc	*Equalvarcov*	*Equalvarcov*	*1*	*1*	*-80.71*	*-80.33*	*172.55*	*171.81*
	Diagonal and equal	Diagonal and equal	1	1	-82.02	-81.84	172.78	172.42
	Equalvarcov	Equalvarcov	2	2	-80.71	-80.34	177.61	176.87
	Diagonal and unequal	Diagonal and unequal	1	1	-82.01	-81.77	177.64	177.15
	Diagonal and equal	Diagonal and equal	2	2	-82.02	-81.84	177.65	177.30
	Diagonal and equal	Equalvarcov	3	3	-82.03	-80.33	180.23	179.56
	Equalvarcov	Diagonal and equal	3	3	-80.71	-81.84	180.29	179.88
	Unconstrained	Unconstrained	1	1	-80.46	-79.18	182.60	180.05
	Diagonal and unequal	Diagonal and unequal	2	2	-82.01	-81.77	182.90	182.42
	Diagonal and unequal	Diagonal and unequal	3	3	-82.01	-81.78	185.69	185.21
	Unconstrained	Unconstrained	2	2	-80.46	-79.35	188.55	186.32
	Unconstrained	Unconstrained	3	3	-80.45	-79.34	191.71	189.48
Gm	*Equalvarcov*	*Equalvarcov*	*1*	*1*	*-71.48*	*-72.36*	*154.25*	*156.01*
	Diagonal and equal	Diagonal and equal	1	1	-72.98	-73.65	154.79	156.15
	Equalvarcov	Diagonal and equal	2	2	-71.48	-73.66	159.46	161.14
	Diagonal and unequal	Equalvarcov	1	2	-72.87	-72.36	159.58	161.23
	Diagonal and equal	Diagonal and unequal	2	1	-72.97	-73.81	159.78	161.46
	Equalvarcov	Diagonal and equal	3	3	-71.49	-73.65	162.25	163.80
	Diagonal and equal	Equalvarcov	3	3	-72.98	-72.37	162.45	164.01
	Diagonal and unequal	Unconstrained	2	1	-72.87	-72.30	165.02	166.78
	Unconstrained	Diagonal and unequal	1	2	-71.90	-73.96	165.99	167.19
	Diagonal and unequal	Diagonal and unequal	3	3	-72.87	-73.81	167.93	169.82
	Unconstrained	Unconstrained	2	2	-70.66	-71.70	169.77	171.84
	Unconstrained	Unconstrained	3	3	-70.67	-71.69	173.13	175.20
Es	*Equalvarcov*	*Diagonal and equal*	*2*	*1*	*-73.59*	*-79.08*	*163.38*	*166.90*
	Diagonal and equal	Equalvarcov	2	1	-75.02	-78.11	163.67	167.35
	Diagonal and unequal	Diagonal and unequal	1	1	-75.12	-78.60	163.86	170.82
	Equalvarcov	Unconstrained	1	1	-76.49	-75.11	164.12	171.89
	Unconstrained	Equalvarcov	1	2	-71.31	-78.11	164.28	172.41
	Diagonal and equal	Diagonal and equal	1	2	-77.82	-79.62	164.39	172.86
	Diagonal and unequal	Equalvarcov	2	3	-73.40	-78.12	165.68	175.10
	Equalvarcov	Diagonal and equal	3	3	-73.59	-79.62	166.06	175.43
	Diagonal and equal	Diagonal and unequal	3	2	-75.02	-79.53	166.24	177.94
	Diagonal and unequal	Unconstrained	3	2	-73.40	-75.17	168.47	177.96
	Unconstrained	Diagonal and unequal	3	3	-71.30	-79.53	170.23	180.73
	Unconstrained	Unconstrained	3	3	-71.31	-75.14	173.39	181.07

Table S7. Results from dynamic factor analysis (DFA) model fitting applied to density (DE) and standardized biomass (BI) indices for batoids by depth stratum (B: 50-100 m; C: 101-200 m; D: 201-500 m) during the periods 1994-2015 in GSA01(Northern Alboran Sea) and GSA06 (Northern Spain) and 2001-2015 in GSA05 (Balearic Islands). For each model, the covariance matrix structure, the number of common trends (m), and the corrected Akaike Information Criterion (AICc) are given. Models in italic represent those selected as the models that produced the most favorable covariance matrix structure (with the lowest AICc values).

**Strata**	**Covariance matrix structure**		**m**		**Log-likelihood**		**AICc**	
	DE	BI	DE	BI	DE	BI	DE	BI
Stratum B	*Diagonal and equal*	*Diagonal and equal*	*1*	*1*	*-77.16*	*-75.46*	*163.05*	*159.67*
	Equalvarcov	Equalvarcov	1	1	-76.51	-74.78	164.16	160.69
	Diagonal and unequal	Diagonal and unequal	1	1	-76.24	-74.92	166.10	163.47
	Diagonal and equal	Diagonal and equal	2	2	-77.15	-75.46	167.92	164.54
	Equalvarcov	Equalvarcov	2	2	-76.52	-74.78	169.23	165.76
	Unconstrained	Diagonal and equal	1	3	-73.98	-75.46	169.63	167.12
	Diagonal and equal	Equalvarcov	3	3	-74.78	-74.78	170.50	168.44
	Diagonal and unequal	Unconstrained	2	1	-76.24	-73.51	171.36	168.70
	Equalvarcov	Diagonal and unequal	3	2	-76.51	-74.91	171.91	168.72
	Diagonal and unequal	Diagonal and unequal	3	3	-76.23	-74.93	174.15	171.52
	Unconstrained	Unconstrained	2	2	-73.98	-73.51	175.57	174.64
	Unconstrained	Unconstrained	3	3	-73.99	-75.51	178.74	177.81
Stratum C	*Diagonal and equal*	*Diagonal and equal*	*1*	*1*	*-80.69*	*-80.25*	*170.13*	*169.26*
	Equalvarcov	Equalvarcov	1	1	-80.05	-79.95	171.24	171.03
	Diagonal and unequal	Diagonal and unequal	1	1	-80.20	-79.64	174.02	172.90
	Diagonal and equal	Diagonal and equal	2	2	-80.76	-80.27	175.15	174.16
	Equalvarcov	Equalvarcov	2	2	-80.05	-79.95	176.30	176.10
	Diagonal and equal	Diagonal and equal	3	3	-80.69	-80.26	177.59	176.71
	Equalvarcov	Diagonal and unequal	3	2	-80.04	-79.65	178.98	178.19
	Diagonal and unequal	Equalvarcov	2	3	-80.29	-79.95	179.47	178.78
	Unconstrained	Unconstrained	1	1	-79.39	-78.72	180.46	179.11
	Diagonal and unequal	Diagonal and unequal	3	3	-80.20	-79.64	182.08	180.95
	Unconstrained	Unconstrained	2	2	-79.43	-78.72	186.49	185.06
	Unconstrained	Unconstrained	3	3	-79.39	-78.73	189.57	188.23
Stratum D	*Equalvarcov*	*Diagonal and equal*	*1*	*1*	*-79.61*	*-80.82*	*170.36*	*170.37*
	Diagonal and equal	Equalvarcov	1	1	-80.86	-79.91	170.46	170.96
	Diagonal and unequal	Diagonal and unequal	1	1	-80.13	-80.52	173.89	174.66
	Diagonal and equal	Diagonal and equal	2	2	-77.86	-80.81	175.34	175.24
	Unconstrained	Equalvarcov	1	2	-76.87	-79.91	175.40	176.02
	Equalvarcov	Diagonal and equal	2	3	-79.62	-80.82	175.42	177.83
	Diagonal and equal	Equalvarcov	3	3	-80.86	-79.91	177.92	178.70
	Equalvarcov	Unconstrained	3	1	-79.62	-78.53	178.11	178.74
	Diagonal and unequal	Diagonal and unequal	2	2	-80.14	-80.52	179.15	179.92
	Unconstrained	Diagonal and unequal	2	3	-76.87	-80.53	181.35	182.71
	Diagonal and unequal	Unconstrained	3	2	-80.13	-78.54	181.94	184.68
	Unconstrained	Unconstrained	3	3	-76.86	-78.51	184.51	187.85

Table S8. Results from dynamic factor analysis (DFA) model fitting applied to density (DE) and standardized biomass (BI) indices for *Torpedo marmorata* during the period 1994-2015 in GSA01(Northern Alboran Sea) and GSA06 (Northern Spain). For each model, the covariance matrix structure, the number of common trends (m), and the corrected Akaike Information Criterion (AICc) are given. Models in italic represent those selected as the models that produced the most favorable covariance matrix structure (with the lowest AICc values).

**Species**	**Error matrix structure**		**M**		**logLik**		**AICc**	
	DE	BI	DE	BI	DE	BI	DE	BI
Torpedo marmorata	*Diagonal and equal*	*Diagonal and equal*	*1*	*1*	*-61.41*	*-61.53*	*129.41*	*129.65*
	Equalvarcov	Equalvarcov	1	1	-61.38	-60.84	131.79	130.72
	Diagonal and equal	Diagonal and unequal	2	1	-61.40	-61.49	131.84	132.01
	Diagonal and unequal	Diagonal and equal	1	2	-61.41	-61.52	131.85	132.10
	Equalvarcov	Equalvarcov	2	2	-61.39	-60.85	134.35	133.27
	Unconstrained	Unconstrained	1	1	-61.38	-60.84	134.37	133.30
	Diagonal and unequal	Diagonal and unequal	2	2	-61.41	-61.50	134.40	134.55
	Unconstrained	Unconstrained	2	2	-61.40	-60.45	137.04	135.97

Table S9. Results from dynamic factor analysis (DFA) model fitting applied to density of size structure by length categories of *Scyliorhinus canicula* during the periods 2000-2015 in GSA01 (Northern Alboran Sea), GSA05 (Balearic Islands) and GSA06 (Northern Spain). For each model, the covariance matrix structure, the number of common trends (m) and the corrected Akaike Information Criterion (AICc) are given. Models in italics represent those selected as the models that produced the most favorable covariance matrix structure (with the lowest AICc values).

Length category	Covariance matrix structure	m	logLik	AICc
< 20 cm	*Diagonal and unequal*	*2*	*-50.96*	*121.70*
	Diagonal and equal	1	-57.03	123.01
	Equalvarcov	1	-55.87	123.20
	Diagonal and unequal	3	-50.95	124.78
	Diagonal and unequal	1	-55.64	125.38
	Unconstrained	1	-52.19	127.25
	Diagonal and equal	2	-57.25	128.60
	Diagonal and equal	3	-56.48	129.84
	Unconstrained	2	-50.98	170.33
	Equalvarcov	2	-57.17	131.22
	Equalvarcov	3	-56.25	132.28
	Unconstrained	3	-50.52	134.22
20–30 cm	*Diagonal and unequal*	*1*	*-54.61*	*123.22*
	Diagonal and equal	1	-60.95	130.86
	Diagonal and unequal	2	-55.68	131.16
	Unconstrained	1	-54.35	131.57
	Diagonal and unequal	3	-54.59	132.06
	Equalvarcov	1	-60.95	133.37
	Diagonal and equal	2	-60.95	136.01
	Unconstrained	3	-54.16	141.49
	Diagonal and equal	3	-62.61	142.09
	Equalvarcov	2	-63.29	143.45
	Equalvarcov	3	-62.48	144.74
	Unconstrained	2	-57.71	144.95
30–40 cm	*Diagonal and unequal*	*1*	*-53.24*	*120.58*
	Unconstrained	1	-51.97	126.81
	Diagonal and equal	1	-59.20	127.34
	Diagonal and unequal	2	-53.95	127.69
	Equalvarcov	1	-58.89	129.24
	Diagonal and unequal	3	-53.25	129.33
	Diagonal and equal	2	-59.57	133.26
	Unconstrained	2	-52.78	135.10
	Equalvarcov	2	-59.42	135.72
	Unconstrained	3	-53.20	139.58
	Diagonal and equal	3	-64.14	145.16
	Equalvarcov	3	-64.03	147.85
40-50 cm	*Diagonal and unequal*	*1*	*-55.62*	*125.33*
	Unconstrained	1	-51.78	126.43
	Diagonal and unequal	2	-54.89	129.58
	Diagonal and equal	2	-59.48	133.06
	Diagonal and unequal	3	-55.10	133.07
	Unconstrained	2	-51.78	133.10
	Diagonal and equal	1	-62.65	134.25
	Equalvarcov	2	-58.73	134.34
	Diagonal and equal	3	-59.48	135.83
	Unconstrained	3	-52.02	137.22
	Equalvarcov	3	-59.05	137.88
	Equalvarcov	1	-65.01	141.63
	Equalvarcov	*1*	*-62.61*	*136.69*
	Diagonal and equal	1	-64.80	138.55
	Equalvarcov	2	-62.62	142.09
	Diagonal and unequal	1	-64.70	143.51

> 50 cm	Diagonal and equal	2	-64.79	143.69
	Equalvarcov	3	-62.62	145.01
	Diagonal and equal	3	-64.79	146.46
	Unconstrained	1	-62.06	146.98
	Diagonal and unequal	2	-64.70	149.19
	Diagonal and unequal	3	-64.70	152.27
	Unconstrained	2	-62.05	153.66
	Unconstrained	3	-62.06	157.29

Table 10. Results from dynamic factor analysis (DFA) model fitting applied to density of size structure by length categories of *Galeus melastomus* during the periods 2000-2015 in GSA01 (Northern Alboran Sea), GSA05 (Balearic Islands) and GSA06 (Northern Spain). For each model, the covariance matrix structure, the number of common trends (m) and the corrected Akaike Information Criterion (AICc) are given. Models in italics represent those selected as the models that produced the most favorable covariance matrix structure (with the lowest AICc values).

Length category	Covariance matrix structure	m	logLik	AICc
< 20 cm	*Diagonal and equal*	*1*	*-65.34*	*139.64*
	Diagonal and unequal	1	-65.09	144.28
	Diagonal and equal	2	-65.34	144.79
	Equalvarcov	1	-67.17	145.80
	Equalvarcov	2	-64.88	146.63
	Diagonal and equal	3	-65.34	147.56
	Equalvarcov	3	-64.88	149.55
	Diagonal and unequal	2	-65.09	149.97
	Unconstrained	1	-64.67	152.20
	Diagonal and unequal	3	-65.09	153.05
	Unconstrained	2	-65.98	161.52
	Unconstrained	3	-64.67	162.51
20–30 cm	*Diagonal and equal*	*1*	*-62.91*	*134.77*
	Diagonal and unequal	1	-61.28	136.67
	Equalvarcov	1	-63.05	137.57
	Diagonal and equal	2	-62.91	139.92
	Diagonal and unequal	2	-61.28	142.35
	Diagonal and equal	3	-62.91	142.69
	Equalvarcov	2	-63.81	144.49
	Unconstrained	1	-60.93	144.72
	Diagonal and unequal	3	-61.28	145.43
	Equalvarcov	3	-63.01	145.81
	Unconstrained	2	-60.87	151.28
	Unconstrained	3	-60.88	154.92
30–40 cm	*Diagonal and unequal*	*1*	*-54.45*	*123.00*
	Unconstrained	1	-50.26	123.39
	Diagonal and equal	1	-59.91	128.78
	Equalvarcov	1	-58.88	129.21
	Unconstrained	2	-50.20	129.95
	Diagonal and unequal	2	-55.37	130.52
	Diagonal and equal	2	-59.91	133.93
	Diagonal and unequal	3	-55.61	134.08
	Equalvarcov	2	-58.88	134.63
	Unconstrained	3	-51.13	135.44
	Diagonal and equal	3	-61.01	138.91
	Equalvarcov	3	-61.02	141.82
40-50 cm	*Diagonal and equal*	*1*	*-61.96*	*132.88*
	Equalvarcov	1	-61.95	135.37
	Diagonal and unequal	1	-60.89	135.89
	Diagonal and equal	2	-61.96	138.02
	Equalvarcov	2	-61.95	140.78
	Diagonal and equal	3	-61.96	140.79
	Unconstrained	1	-59.16	141.18
	Diagonal and unequal	2	-60.89	141.58
	Equalvarcov	3	-61.95	143.69
	Diagonal and unequal	3	-60.89	144.65
	Unconstrained	2	-59.16	147.86
	Unconstrained	3	-59.17	151.49
	Diagonal and equal	*1*	*-62.35*	*133.65*
	Diagonal and unequal	1	-60.59	135.28
	Equalvarcov	2	-62.17	135.81
	Diagonal and equal	2	-62.34	138.79

> 50 cm	Diagonal and unequal	2	-60.58	140.96
	Equalvarcov	2	-62.17	141.22
	Diagonal and equal	3	-62.17	141.56
	Unconstrained	1	-59.48	141.83
	Diagonal and unequal	3	-60.58	144.04
	Equalvarcov	3	-62.17	144.13
	Unconstrained	2	-59.48	148.51
	Unconstrained	3	-59.49	152.15

Table S11. Results from dynamic factor analysis (DFA) model fitting applied to density of size structure by length categories of *Etmopterus spinax* during the periods 2000-2015 in GSA01 (Northern Alboran Sea), GSA05 (Balearic Islands) and GSA06 (Northern Spain). For each model, the covariance matrix structure, the number of common trends (m) and the corrected Akaike Information Criterion (AICc) are given. Models in italics represent those selected as the models that produced the most favorable covariance matrix structure (with the lowest AICc values).

Length category	Covariance matrix structure	m	logLik	AICc
< 15 cm	*Diagonal and unequal*	*1*	*-38.97*	*93.19*
	Equalvarcov	1	-41.18	94.59
	Diagonal and equal	1	-43.67	96.77
	Diagonal and unequal	2	-38.98	99.95
	Equalvarcov	2	-41.18	100.84
	Diagonal and equal	2	-43.63	102.49
	Unconstrained	1	-38.52	102.88
	Diagonal and unequal	3	-38.98	103.78
	Equalvarcov	3	-41.18	104.36
	Diagonal and equal	3	-43.55	105.58
	Unconstrained	2	-38.52	111.61
	Unconstrained	3	-38.53	116.65
15–25 cm	*Equalvarcov*	*1*	*-40.87*	*93.97*
	Diagonal and equal	1	-43.24	95.90
	Diagonal and unequal	1	-40.58	96.40
	Diagonal and equal	2	-41.55	98.33
	Diagonal and equal	3	-41.54	101.56
	Equalvarcov	2	-41.63	101.74
	Diagonal and unequal	2	-40.96	103.92
	Equalvarcov	3	-41.51	105.02
	Unconstrained	1	-40.53	106.89
	Diagonal and unequal	3	-40.95	107.73
	Unconstrained	2	-40.25	115.08
	Unconstrained	3	-40.23	120.08
25–35 cm	*Diagonal and equal*	*1*	*-44.82*	*99.07*
	Diagonal and unequal	1	-42.10	99.43
	Equalvarcov	1	-43.81	99.86
	Unconstrained	1	-37.40	100.63
	Diagonal and equal	2	-44.81	104.86
	Equalvarcov	2	-43.82	106.12
	Diagonal and unequal	2	-42.07	106.13
	Unconstrained	2	-37.38	109.32
	Equalvarcov	3	-43.82	109.64
	Diagonal and unequal	3	-42.07	109.96
	Unconstrained	3	-37.34	114.29
	Diagonal and equal	3	-48.17	114.82
> 35 cm	*Diagonal and equal*	*1*	*-43.73*	*96.88*
	Equalvarcov	1	-43.63	99.49
	Diagonal and unequal	1	-42.34	99.91
	Diagonal and equal	2	-43.33	101.88
	Equalvarcov	2	-43.14	104.76
	Diagonal and equal	3	-43.31	105.12
	Diagonal and unequal	2	-42.07	106.14
	Unconstrained	1	-40.82	107.46
	Equalvarcov	3	-43.10	108.20
	Diagonal and unequal	3	-42.03	109.89
	Unconstrained	2	-40.29	115.14
	Unconstrained	3	-40.27	120.14

Table S12. Length at first maturity (L_{50}; or length in cm TL at which 50% of specimens had become mature), logistic regression coefficients of maturity ogive (α and β) and the smallest mature female (SMF; cm TL) of *Scyliorhinus canicula* per year and sex (F: female; M: males) in the geographical sub-areas considered by the General Fisheries Commission for the Mediterranean in the study area (GSA01: Northern Alboran Sea; GSA05: Balearic Islands; GSA06: Northern Spain) during the periods 2000-2015 in GSA01 and GSA06 and 2007-2015 in GSA05. n: number of samples analysed. Statistical significance: (*) $p<0.05$; (**) $p<0.01$; (***) $p<0.001$.

GSAs	Years	n		L_{50}		α		β		SMF
		F	M	F	M	F	M	F	M	
GSA01	2000	56	52	45.54	41.17	-51.81**	-15.09**	0.11**	0.03***	44
	2001	64	62	44.99	42.14	-38.46**	-25.52*	0.08**	0.06*	43
	2002	205	332	45.62	41.20	-27.18**	-22.22***	0.06**	0.05***	44
	2003	163	202	45.99	41.22	-33.45***	-25.30***	0.07***	0.06***	43.5
	2004	93	104	45.13	41.60	-22.04***	-13.03***	0.04***	0.03***	41.5
	2005	114	148	42.59	41.72	-25.12***	-19.65***	0.05***	0.04***	41
	2006	164	254	43.92	41.94	-26.85***	-45.46***	0.06***	0.10***	40.5
	2007	240	292	43.92	41.11	-30.96***	-34.20***	0.07***	0.08***	41
	2008	125	151	42.14	41.90	-25.54***	-26.78***	0.06***	0.06***	39
	2009	104	146	42.47	40.63	-23.03***	-20.01***	0.05***	0.04***	38
	2010	51	60	42.15	41.28	-27.30*	-14.87**	0.06*	0.03**	38
	2011	63	61	42.70	40.13	-31.26**	-37.24**	0.07**	0.09**	39
	2012	87	101	41.52	39.72	-25.04**	-16.20***	0.06**	0.04***	37.5
	2013	149	138	42.20	39.30	-22.50**	-25.10**	0.06**	0.06**	38
	2014	98	97	40.90	38.95	-34.31***	-24.42***	0.08***	0.06***	38.5
	2015	299	316	40.61	39.69	-26.92***	-22.74***	0.06***	0.06***	37
GSA05	2007	670	917	41.19	40.98	-23.72***	-20.54***	0.06***	0.05***	38
	2008	698	869	41.44	40.25	-21.42***	-22.52***	0.05***	0.05***	37.5
	2009	722	890	40.84	40.05	-25.20***	-43.43***	0.06***	0.10***	37.5
	2010	785	925	40.75	40.01	-24.26***	-50.30***	0.06***	0.12***	36.5
	2011	626	854	40.64	39.87	-17.18***	-19.78***	0.04***	0.02***	37
	2012	246	362	39.98	39.38	-12.44***	-11.75***	0.03***	0.03***	37.5
	2013	282	388	40.60	39.20	-23.40**	-24.10**	0.05**	0.06**	37
	2014	568	743	39.96	38.26	-22.20***	-16.87***	0.05***	0.04***	36.5
	2015	729	827	39.45	38.30	-26.84***	-13.22***	0.06***	0.03***	36
GSA06	2000	247	307	43.37	41.24	-19.85***	-51.32***	0.04***	0.12***	41.5
	2001	402	296	44.66	40.83	-29.12***	-20.42***	0.06***	0.05***	41.5
	2002	372	509	44.59	41.06	-19.30***	-25.97***	0.04***	0.06***	39.5
	2003	363	370	44.48	41.12	-15.51***	-18.96***	0.03***	0.04***	39
	2004	254	366	43.83	41.22	-19.61***	-15.67***	0.04***	0.03***	39.5
	2005	555	632	42.66	41.72	-21.03***	-14.23***	0.05***	0.03***	38
	2006	295	421	41.77	41.63	-19.97***	-14.17**	0.04***	0.03**	39
	2007	412	463	42.08	41.64	-22.10***	-20.29***	0.05***	0.04***	38
	2008	267	346	42.68	41.73	-29.37***	-21.03***	0.06***	0.05***	39.5
	2009	282	351	42.10	41.07	-30.84**	-20.67***	0.07**	0.05***	39.5
	2010	235	259	40.98	39.05	-17.81***	-30.49***	0.04***	0.07***	38
	2011	318	386	41.86	40.86	-18.28***	-38.04***	0.04***	0.09***	37.5
	2012	442	476	41.37	40.30	-34.11***	-29.65**	0.08***	0.07**	37.5
	2013	393	433	40.90	39.60	-26.00**	-17.90**	0.06**	0.04**	36
	2014	330	382	39.97	39.19	-28.17***	-26.61***	0.07***	0.06***	37
	2015	534	539	40.04	38.74	-21.08***	-18.14***	0.05***	0.04***	36.5

Table S13. Length at first maturity (L_{50}; or length in cm TL at which 50% of specimens had become mature), logistic regression coefficients of maturity ogive (α and β) and the smallest mature female (SMF; cm TL) of *Galeus melastomus* per year and sex (F: female; M: males) in the geographical sub-areas considered by the General Fisheries Commission for the Mediterranean in the study area (GSA01: Northern Alboran Sea; GSA05: Balearic Islands; GSA06: Northern Spain) during the periods 2000-2015 in GSA01 and GSA06 and 2007-2015 in GSA05. n: number of samples analysed. Statistical significance: (*) p<0.05; (**) p<0.01; (***) p<0.001.

GSAs	Years	n		L_{50}		α		β		SMF
		F	M	F	M	F	M	F	M	
GSA01	2000	168	241	53.21	48.59	-23.30***	-18.54***	0.04***	0.03***	50.5
	2001	264	295	54.04	49.94	-29.20***	-44.63***	0.05***	0.08***	51
	2002	247	348	54.11	50.17	-50.26**	-40.57***	0.09**	0.08***	51
	2003	270	384	53.26	49.55	-37.78***	-44.20***	0.07***	0.08***	48.5
	2004	182	185	53.02	48.09	-22.50**	-29.30***	0.04**	0.06***	48.5
	2005	196	348	52.90	48.49	-22.01***	-37.20***	0.04***	0.07***	49
	2006	401	567	53.45	48.70	-31.52***	-27.45***	0.05***	0.05***	49.5
	2007	260	329	52.93	48.62	-31.91***	-46.90***	0.06***	0.09***	49
	2008	225	302	53.29	48.41	-39.64***	-27.51***	0.07***	0.05***	50
	2009	270	323	53.46	48.39	-36.46***	-42.85***	0.06***	0.08***	51
	2010	124	129	53.11	48.64	-27.72***	-27.31***	0.05***	0.05***	49
	2011	121	150	53.57	48.18	-29.21**	-28.86***	0.05**	0.05***	50
	2012	248	326	53.54	47.90	-27.63***	-27.19***	0.05**	0.05***	51
	2013	446	481	54.10	47.20	-25.90*	-35.80*	0.05*	0.07*	51
	2014	219	201	53.38	48.28	-20.29***	-36.12***	0.03***	0.07***	49
	2015	389	482	52.94	47.21	-22.91***	-37.24***	0.04***	0.07***	49.5
GSA05	2007	359	438	50.58	49.08	-19.69***	-29.49***	0.03***	0.06***	50
	2008	319	323	50.31	48.39	-17.28**	-14.27***	0.03**	0.02***	49
	2009	215	254	51.86	50.22	-14.66***	-25.57**	0.02***	0.05**	48.5
	2010	264	317	51.51	49.89	-34.90**	-33.31***	0.06**	0.06***	49.5
	2011	147	270	51.07	49.53	-23.02***	-24.21***	0.04***	0.04***	50.5
	2012	191	317	52.22	48.97	-32.63*	-24.97***	0.06*	0.05***	50
	2013	136	143	52.10	49.90	-50.90*	-31.30*	0.09*	0.06*	51
	2014	266	301	51.33	48.99	-20.33*	-17.22**	0.03*	0.03**	49.5
	2015	854	448	50.89	48.74	-16.52***	-23.00***	0.03***	0.04***	49
GSA06	2000	195	216	53.81	50.04	-49.54**	-34.54***	0.09**	0.06***	51
	2001	394	413	53.66	49.77	-19.76**	-23.37*	0.03**	0.04*	49.5
	2002	321	431	54.14	49.91	-43.09**	-37.81***	0.07**	0.07***	51
	2003	145	145	53.94	48.81	-22.83*	-25.55***	0.04*	0.05***	50.5
	2004	262	271	53.32	48.89	-45.55***	-30.13***	0.08***	0.06***	51
	2005	319	286	52.55	50.00	-29.05***	-47.18***	0.05***	0.09***	50
	2006	346	431	51.66	49.12	-23.23***	-51.22***	0.04***	0.10***	49
	2007	424	463	52.73	49.00	-30.39***	-46.21***	0.05***	0.09***	49.5
	2008	112	91	53.05	48.06	-36.84*	-23.16**	0.06*	0.04**	50.5
	2009	141	150	52.77	47.95	-29.72**	-36.20**	0.05**	0.07**	49
	2010	86	115	53.04	48.41	-22.15**	-46.46***	0.04**	0.09***	49
	2011	197	198	53.37	49.61	-42.21***	-30.07***	0.07***	0.06***	49.5
	2012	366	385	52.18	49.22	-37.99**	-28.34***	0.07**	0.05***	48.5
	2013	210	214	52.80	49.30	-34.50*	-39.70*	0.06*	0.08*	47
	2014	192	213	51.97	48.70	-32.79***	-42.44***	0.06***	0.08***	48
	2015	229	263	51.92	49.91	-31.12***	-37.81***	0.05***	0.07***	48.5

Table S14. Length at first maturity (L_{50}; or length in cm TL at which 50% of specimens had become mature), logistic regression coefficients of maturity ogive (α and β) and the smallest mature female (SMF; cm TL) of *Raja clavata* per year and sex (F: female; M: males) in the GSA05 (Balearic Islands) during the period 2007-2015. n: number of samples analysed. Statistical significance: (*) p<0.05; (**) p<0.01; (***) p<0.001.

**Years**	**n**		**L_{50}**		**α**		**β**		**SMF**
	**F**	**M**	**F**	**M**	**F**	**M**	**F**	**M**	
2007	107	158	75.15	68.00	-62.63*	-21.98***	0.08*	0.03***	73
2008	69	86	75.48	67.71	-15.44**	-21.22**	0.02**	0.03**	73.5
2009	94	83	77.02	66.99	-39.03*	-42.05**	0.05*	0.06**	74
2010	93	100	75.67	69.16	-18.47***	-18.46***	0.02***	0.02***	73.5
2011	93	74	77.70	66.29	-30.13	-16.27***	0.03**	0.02***	74
2012	88	70	75.90	66.41	-27.84*	-17.61**	0.03*	0.02**	73.5
2013	71	79	78.10	68.60	-21.30*	-13.10*	-0.03*	0.02*	75
2014	113	120	76.88	66.52	-53.30*	-17.34***	0.06*	0.02***	73.5
2015	82	73	76.09	66.32	-24.59**	-24.08***	0.03**	0.03***	74
